# Developmental vascular malformations in *EPAS1* gain-of-function syndrome

**DOI:** 10.1172/jci.insight.144368

**Published:** 2021-03-08

**Authors:** Jared S. Rosenblum, Herui Wang, Pauline M. Dmitriev, Anthony J. Cappadona, Panagiotis Mastorakos, Chen Xu, Abhishek Jha, Nancy Edwards, Danielle R. Donahue, Jeeva Munasinghe, Matthew A. Nazari, Russell H. Knutsen, Bruce R. Rosenblum, James G. Smirniotopoulos, Alberto Pappo, Robert F. Spetzler, Alexander Vortmeyer, Mark R. Gilbert, Dorian B. McGavern, Emily Chew, Beth A. Kozel, John D. Heiss, Zhengping Zhuang, Karel Pacak

**Affiliations:** 1 Neuro-Oncology Branch, National Cancer Institute, NIH, Bethesda, Maryland, USA.; 2 Surgical Neurology Branch, National Institute of Neurological Disorders and Stroke, NIH, Bethesda, Maryland, USA.; 3 Viral Immunology and Intravital Imaging Section, National Institute of Neurological Disorders and Stroke, NIH, Bethesda, Maryland, USA.; 4 Section on Medical Neuroendocrinology, *Eunice Kennedy Shriver* National Institute of Child Health and Human Development, NIH, Bethesda, Maryland, USA.; 5 Mouse Imaging Facility, National Institute of Neurological Disorders and Stroke, NIH, Bethesda, Maryland, USA.; 6 Internal Medicine and Pediatrics, MedStar Georgetown University Hospital, Washington, DC, USA.; 7 Laboratory of Vascular and Matrix Genetics, National Heart Lung and Blood Institute, NIH, Bethesda, Maryland, USA.; 8 Department of Neurosurgery, Riverview Medical Center, Red Bank, New Jersey, USA.; 9 Department of Radiology, School of Medicine and Health Sciences, George Washington University, Washington, DC, USA.; 10 National Library of Medicine, Bethesda, Maryland, USA.; 11 Oncology Department, Developmental Biology and Solid Tumor Program, St. Jude Comprehensive Cancer Center, St. Jude Children’s Research Hospital, Memphis, Tennessee, USA.; 12 Department of Neurosurgery, Barrow Neurological Institute, St. Joseph’s Hospital, and Medical Center, Phoenix, Arizona, USA.; 13 Department of Pathology and Laboratory Medicine, Indiana University School of Medicine, Indianapolis, Indiana, USA.; 14 Division of Epidemiology and Clinical Applications, National Eye Institute, NIH, Bethesda, Maryland, USA.

**Keywords:** Angiogenesis, Development, Genetic diseases, Mouse models

## Abstract

Mutations in *EPAS1*, encoding hypoxia-inducible factor-2α (HIF-2α), were previously identified in a syndrome of multiple paragangliomas, somatostatinoma, and polycythemia. HIF-2α, when dimerized with HIF-1β, acts as an angiogenic transcription factor. Patients referred to the NIH for new, recurrent, and/or metastatic paraganglioma or pheochromocytoma were confirmed for *EPAS1* gain-of-function mutation; imaging was evaluated for vascular malformations. We evaluated the *Epas1*^A529V^ transgenic syndrome mouse model, corresponding to the mutation initially detected in the patients (*EPAS1*^A530V^), for vascular malformations via intravital 2-photon microscopy of meningeal vessels, terminal vascular perfusion with Microfil silicate polymer and subsequent intact ex vivo 14T MRI and micro-CT, and histologic sectioning and staining of the brain and identified pathologies. Further, we evaluated retinas from corresponding developmental time points (P7, P14, and P21) and the adult dura via immunofluorescent labeling of vessels and confocal imaging. We identified a spectrum of vascular malformations in all 9 syndromic patients and in all our tested mutant mice. Patient vessels had higher variant allele frequency than adjacent normal tissue. Veins of the murine retina and intracranial dura failed to regress normally at the expected developmental time points. These findings add vascular malformation as a new clinical feature of *EPAS1* gain-of-function syndrome.

## Abstract 2

We discovered vascular malformations due to failure of developmental vascular regression in patients with *EPAS1* gain-of-function mutation syndrome and the corresponding transgenic mouse model.

## Graphical abstract

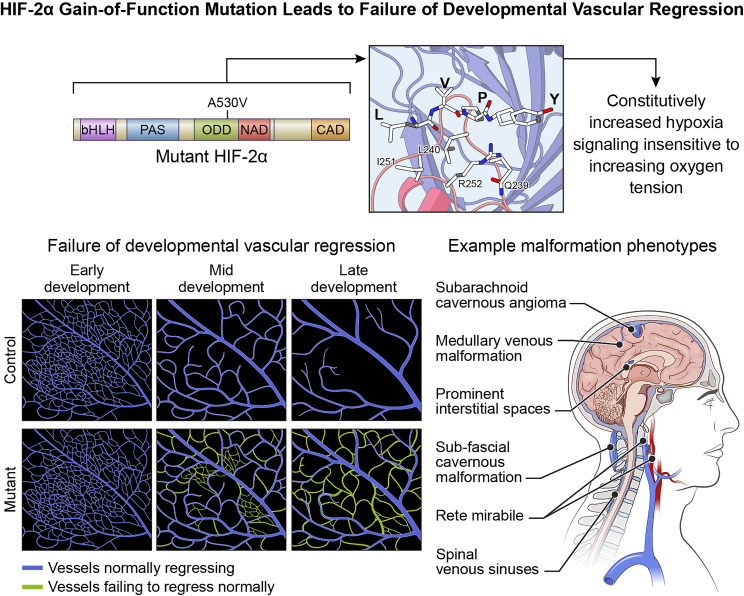

## Introduction

We previously identified the syndrome of multiple paragangliomas, somatostatinoma, and polycythemia resulting from postzygotic *EPAS1* gain-of-function mutations (1). Mutations of the oxygen degradation domain (ODD) of hypoxia-inducible factor-2α (HIF-2α), encoded by *EPAS1*, have been shown to impair hydroxylation by prolyl hydroxylase domain-containing protein 2 (PHD2) and subsequent association with the von Hippel-Lindau (VHL) protein (1–4). Degradation of HIF-2α is impaired, resulting in its stabilization, prolonged activation, and lack of response to normal or increasing oxygen tension (5, 6).

Prior studies have indicated that HIF-2α–mediated signaling modulates angiogenesis and vasculogenesis that develops the arterial and venous systems (7). HIF-2α is activated by tightly regulated regions of hypoxia during vascular development and is highly expressed by endothelial cells (8). HIF-2α signaling has previously been studied by either deletion of HIF-2α or inactivation of PHD2, which is thought to provide gain-of-function of HIF-2α (9–11). However, the HIF subunits can still associate with VHL and be degraded by the proteasome without modification by PHD2, though at a slower rate (12, 13). Thus, this syndrome, in which the ODD of HIF-2α is mutated, is unique in that it is a true gain of function. This allows direct study of the effects of HIF-2α–mediated signaling in vascular development.

Here, we sought to investigate the effect of HIF-2α gain of function on vascular development in *EPAS1* gain-of-function syndrome patients and the corresponding transgenic *Epas1* mouse model.

## Results

### Patient clinical characteristics.

Representative imaging results are provided for 3 index patients. Patient characteristics for all 9 patients are provided in Table 1 and Supplemental Table 1 (supplemental material available online with this article; https://doi.org/10.1172/jci.insight.144368DS1), and additional patient imaging is provided in Supplemental Figures 1–9. Patients were neurologically intact at presentation except for some with documented poor visual acuity from early childhood, such as previously reported in patient 2 (14). Patient 3 complained of chronic central scotoma in the left eye.

### Congenital malformations of the eye in the syndromic patients.

Ophthalmologic evaluation of 8 patients found optic disc elevation without optic cup in 7 patients (Supplemental Figures 1–5, 7, and 8), indicating pseudopapilledema owing to congenital vascular malformation (15, 16). All patients evaluated by fundoscopy had large retinal veins with abnormal branching patterns. Patient 1 demonstrated hemangiomatous lesions arising from postcapillary venules in the peripheral retinal fields bilaterally as previously reported (Supplemental Figure 1); patient 9 had an endophytic juxtapapillary retinal capillary hemangioma (Supplemental Figure 9) (14). Patient 2 had morning glory anomaly of the right eye, persistent fibrovascular hyaloid membrane, and thick choroid with large veins throughout as previously reported (Supplemental Figure 2) (14). Patient 3 showed evidence of partial morning glory anomaly first detected at 18 months old (Supplemental Figure 3). Patients 1–5 and 7–9 had varying degrees of fibrovascular membranes originating from the optic disc (Supplemental Figures 1–5 and 7–9). Patients 2, 3, 7, and 8 demonstrated abnormalities of the macula, with patient 8 having retinal hemorrhage from a central retinal venous malformation. These are often associated with ipsilateral brain vascular malformations (17).

### Systemic venous malformations in syndromic patients.

**Figure 1:**
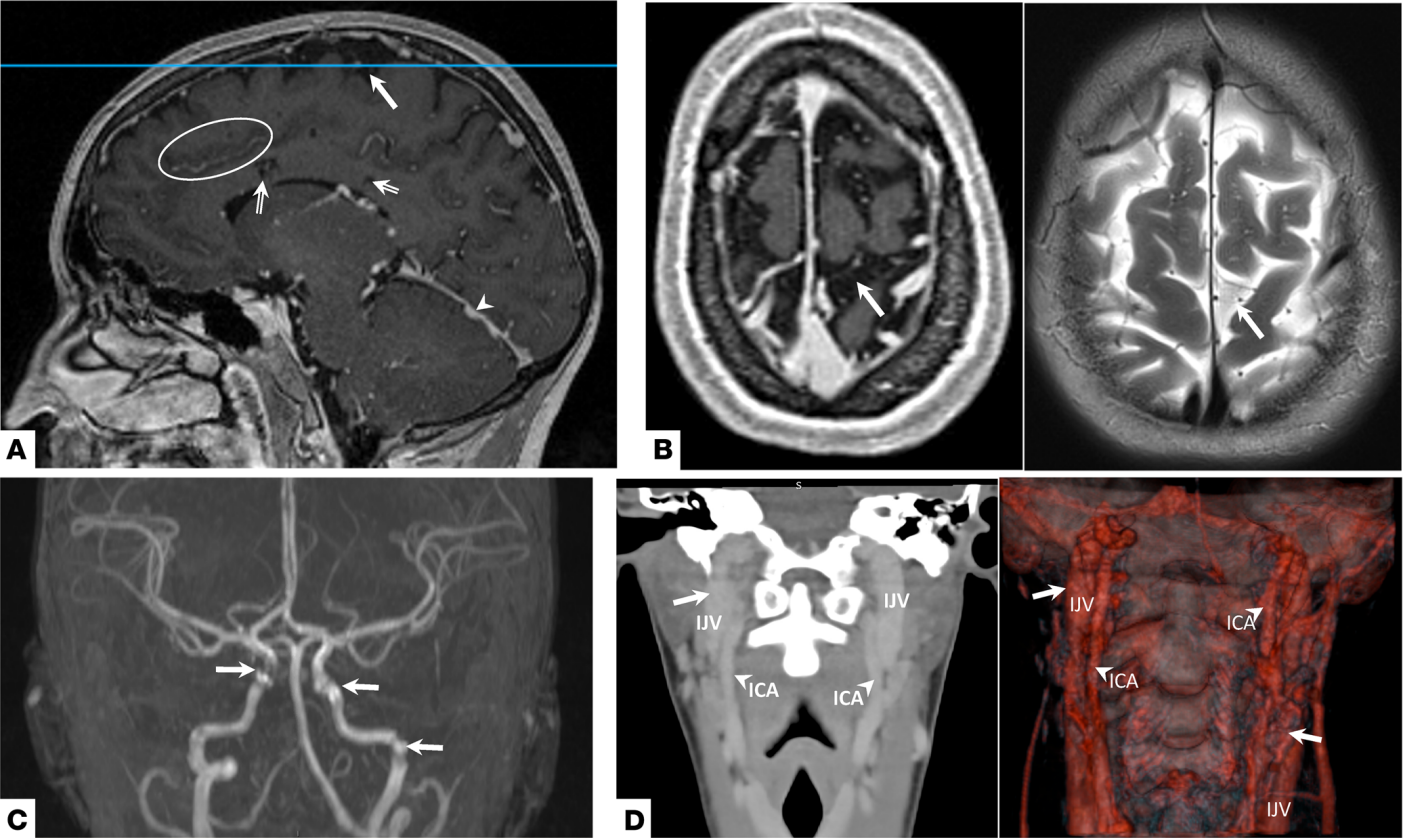
Intracranial malformations and rete mirabile in HIF-2α gain-of-function syndrome patients. Representative images are shown. (**A**) Sagittal T1-weighted postcontrast MRI of the head of patient 1 shows a subarachnoid cavernous angioma (arrow). Prominent interstitial fluid or Virchow-Robins spaces are seen within the corpus callosum (double-lined arrows); these are seen throughout the parenchyma following veins (white circle). Prominent tentorial veins are also seen (arrowhead). (**B**) Axial T1-FLAIR (left) and T2-weighted (right) sequences of the same patient show the subarachnoid cavernous angioma (arrow) arising from veins. This corresponds to the blue line in **A**. (**C**) Reconstruction of TOF MR angiogram of patient 3 shows dysplastic segments of the internal carotid artery bilaterally (arrows). (**D**) Coronal CT of the neck with contrast of the same patient shows a plexiform jugular vein surrounding a dysplastic carotid, known as a rete mirabile, at the cranio-cervical junction on the right (arrow); volumetric reconstruction of the same CT of the neck demonstrating rete mirabile on the right and left (arrows). ICA, internal carotid artery; IJV, internal jugular vein.

MRI of the brain with contrast demonstrated subarachnoid cavernous malformations and prominent interstitial or perivascular spaces throughout the brain in patients 1 (Figure 1, A and B) and 4 (Supplemental Figure 4). Patients 8 and 9 had tentorial-based venous malformations with prominent perivascular spaces (Supplemental Figures 8 and 9). Five patients displayed venous malformations within the quadrigeminal cistern draining to the confluence of the vein of Galen (Supplemental Figures 2, 4–6, and 9), with some involving the pineal gland and tectum. The dural sinuses and confluence of the vein of Galen were prominent in 8 of 9 patients. TOF magnetic resonance angiography of the brain in patient 3 revealed dysplastic segments of the internal carotid artery, indicative of rete mirabile, a plexiform webbing of the internal jugular vein around dysplastic carotid arteries (Figure 1C). Subsequent evaluation of the head and neck vessels with contrasted CT demonstrated rete mirabile of the right internal carotid artery and jugular vein in this patient (Figure 1D). Four additional patients (1, 5, 7, and 9) also had rete mirabile involving the jugular vein (Supplemental Figures 1, 5, 7, and 9).

**Figure 2:**
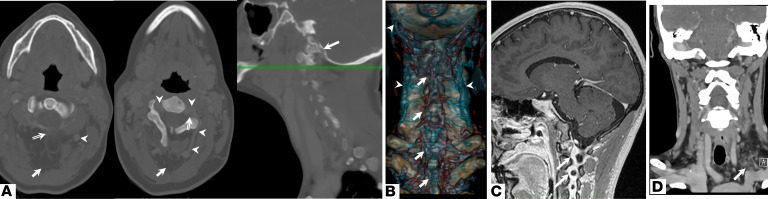
Extrinsic cervical spine venous malformations in HIF-2α gain-of-function syndrome patients. Representative images are shown. (**A**) Axial CT of the neck with contrast of patient 1 shows cervical dysraphism at the level of C2 (left) due to a subfascial cavernous malformation of the upper neck (arrow). The malformation is continuous with the epidural veins (double-lined arrow) and large veins of the external spinal system (arrowhead). At the level of C3–4 (middle), the malformation (arrow) drains to a persistent segmental vein (arrowheads) of the spinal column bilaterally. These surround the vertebral artery (double-lined arrow). Large veins are seen throughout the soft tissues. Sagittal view of the left side of the same CT of the neck (right) showing the corresponding axial plane (green line). The posterior condylar emissary vein (arrow) is also seen draining to the malformation. (**B**) Posterior coronal view of the volumetric reconstruction of CT of the neck with contrast of patient 1 highlighting contrast and bone and excluding soft tissue demonstrates the veins (blue; arrowheads) entering and within the cavernous malformation (arrow) lined by persistent mesenchyme (red outline) extending from beneath the skull base down the length of the cervical spine. (**C**) Sagittal T1-weighted postcontrast MRI of the head demonstrates large sinusoidal veins (arrows) throughout the left lateral aspect of the neck of patient 2. (**D**) Coronal CT of the neck with contrast of patient 1 shows a venous malformation draining to the left jugular and subclavian veins (arrow).

Imaging of the neck also revealed a subfascial cavernous malformation in the posterior aspect of the neck in 7 of 9 patients (Figure 2 and Supplemental Figures 1, 3–5, and 7–9). This malformation is continuous with the cervical epidural veins, the posterior condylar emissary veins, and large anomalous segmental or intervertebral veins (Figure 2, A and B). In 5 patients, the posterior condylar emissary vein was anomalous and disrupted the occipital bone as it drained to prominent cranio-cervical plexuses (Supplemental Figures 1–3, 5, and 9). This was previously reported in patients 1–3 and 5 (18).

The anomalous segmental veins are connected to anomalous large deep cervical veins as seen in volumetric reconstruction (Figure 2B). Patients 2–4 and 7–9 had cervical venous sinuses (Supplemental Figures 2–4 and 7–9) draining anomalous cranio-cervical plexuses into the segmental veins. In 4 patients, these segmental veins drained to prominent anomalous vertebral veins (Supplemental Figures 3, 4, 7, and 9), which subsequently drained to the subclavian vein. Additionally, in 3 patients, a venous malformation arising from the subclavian vein was observed (Figure 2D and Supplemental Figures 5 and 9).

**Figure 3:**
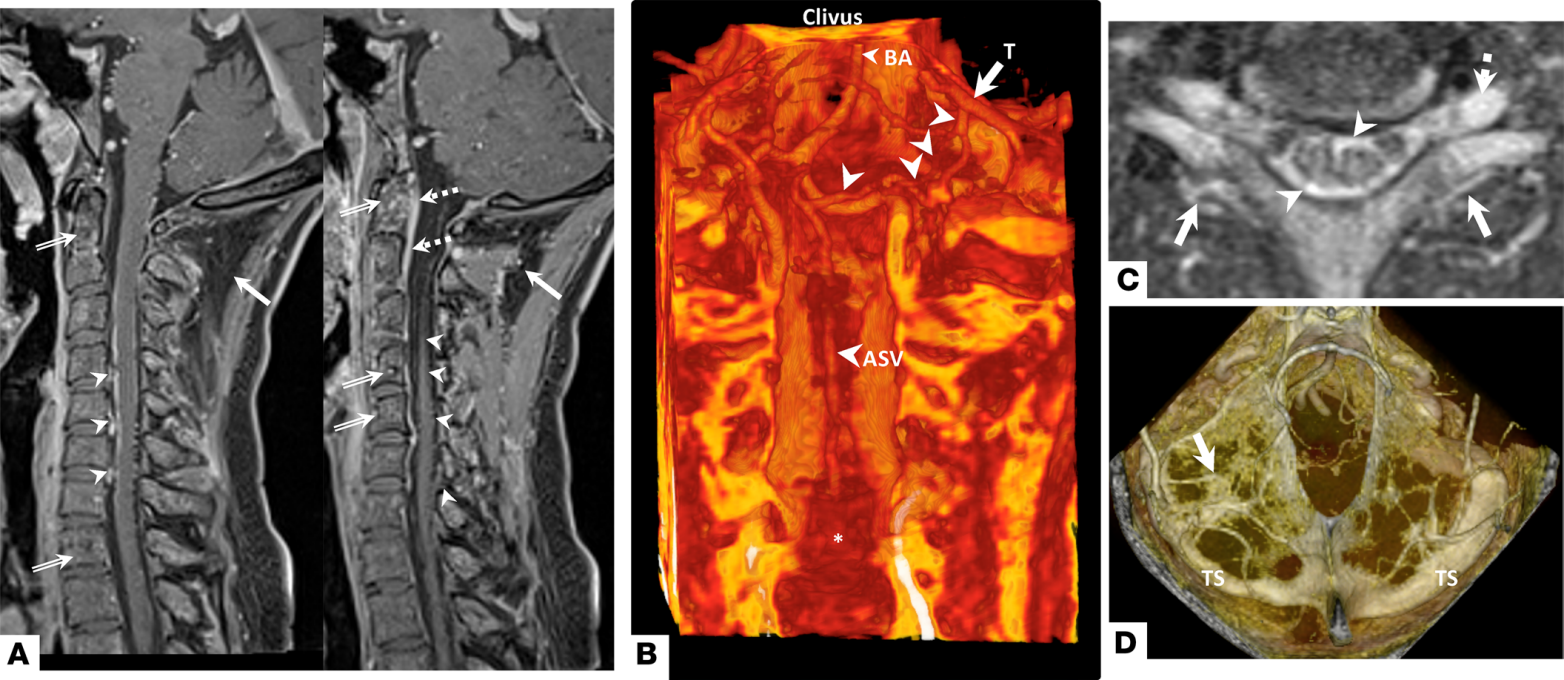
Meningeal and parenchymal venous malformations in HIF-2α gain-of-function syndrome patients. (**A**) Midsagittal T1-weighted MRI of the cervical spine with contrast of patient 1 shows the cavernous malformation of the neck (arrow) and multiple contrast enhancing vessels behind the C4–7 vertebral bodies (arrowheads). Contrast enhancement in abnormal appearing regions of vertebral bodies is also seen (double-lined arrows). Adjacent slice of the same MRI shows contrast enhancement of the meninges connecting to the veins of the malformation (arrowheads) on the left. Abnormal enhancement of the bone and ligaments (double-lined arrows) is found to be continuous with contrast enhancement within the dura posterior to the vertebral bodies (dashed arrows). The neck malformation is again seen (arrow). (**B**) Posterior coronal view of volumetric reconstruction of the same 3D T1-weighted postcontrast sequence MRI excluding parenchyma and highlighting bone and contrast shows a venous malformation (asterisk) originating from the anterior spinal vein (ASV) corresponding to the intradural contrast enhancing vessels in **A**; the ASV is connected (arrowheads) to the tentorium (T). BA, basilar artery. (**C**) Axial T1-weighted postcontrast MRI of the same patient shows the veins in the dural nerve root sleeve (dashed arrow) draining the veins of the spinal cord (arrowheads) with a widened anterior median sulcus; the enlarged external spinal column veins are also seen (arrows). (**D**) Volumetric reconstruction of T1-weighted postcontrast MRI of the head of patient 2 shows a developmental configuration of the tentorial veins and sinuses on the left (arrow); the left transverse sinus (TS) is smaller than the right.

MRI of the neck in patient 1 showed prominent contrast enhancement posterior to the vertebral bodies in the cervical spine (Figure 3A). Volumetric reconstruction demonstrated a venous malformation arising from the anterior spinal vein with an anomalous connection to the tentorial veins (Figure 3B). The leptomeningeal veins of the malformation were also continuous with both of the veins surrounding the nerve root and the intrinsic medullary veins of the spinal cord (Figure 3C). Three patients also demonstrated plexiform anomalous tentorial venous drainage (Figure 3D and Supplemental Figures 7 and 8); for some patients, the MRI slices were too thick to visualize tentorial drainage. Patient 9 had an enlarged central canal of the cervical spinal cord (Supplemental Figure 9), which has been stable over the duration of follow-up.

Volumetric reconstruction of CT of the chest, abdomen, and pelvis with contrast performed for tumor staging and restaging were also evaluated for systemic vascular malformations. The cervical venous malformation in patient 1 was found to be continuous with an anomalous external spinal venous system extending to a dysraphic sacrum (Supplemental Figure 10). Seven patients had similar sacral dysraphism or abnormal segmentation as previously reported (17). Further, in patient 1, an aberrant vein was seen in the thoracolumbar region draining the malformation to the inferior vena cava (Supplemental Figure 10). Patient 3 had prominent lumbar epidural veins draining to plexiform segmental veins, continuing to the inferior vena cava and iliac veins through anomalous lumbar veins (Supplemental Figure 3, A–C). Patient 9 had anomalous drainage of the epidural veins in the thoracolumbar spine to the inferior vena cava (Supplemental Figure 9).

### Vessel allele frequency is greater than in normal tissue.

Variant (P531S) allele frequency quantified in vessels and adjacent normal tissue from tumor specimens of 2 patients by manual microdissection from paraffin-embedded sections and digital droplet PCR (ddPCR) demonstrated that the variant allele frequency in the vessel is higher than in adjacent normal tissue. The allele frequency in the tumor vessels for patient 2 was 9.27%, whereas the adjacent normal tissue was 2.4%; the tumor tissue was 62%. In patient 9, the allele frequency in the tumor vessels was 48%, whereas the adjacent normal tissue was 5%; the tumor tissue was 81%.

### Systemic venous malformations in the syndromic mouse model.

**Figure 4:**
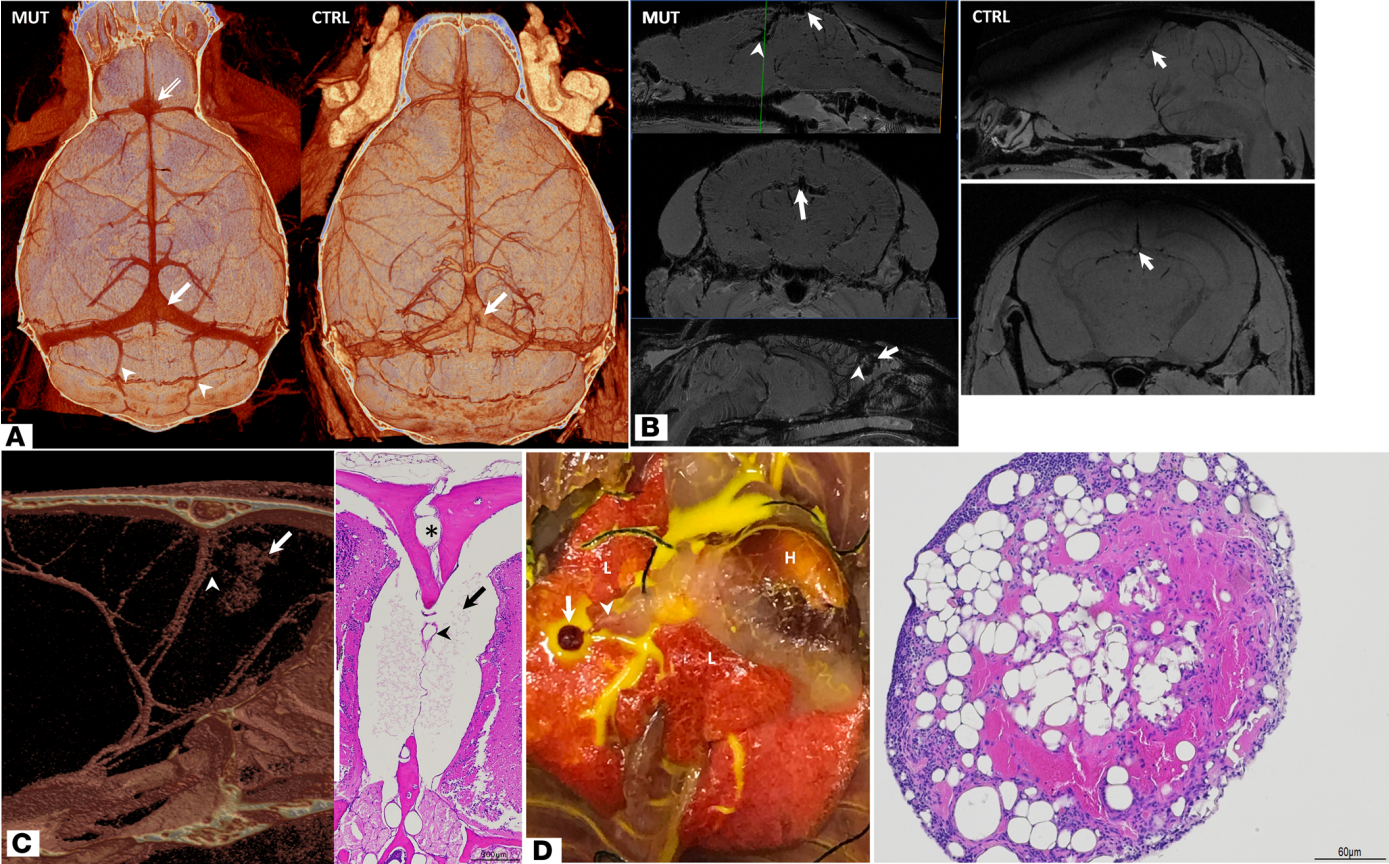
Venous anomalies and malformations in HIF-2α gain-of-function syndrome mouse model. Representative images are shown. (**A**) Axial view 3D volumetric reconstruction of micro-CT of the polymer-casted mouse model (MUT) shows a prominent confluence of sinuses (arrow) compared with control (CTRL) and a vascular malformation arising from the junction of the superior sagittal sinus and rostral rhinal sinus (double-lined arrow). Prominent occipital emissary veins (arrowheads) are seen in the mutant. (**B**) Sagittal ex vivo T1-weighted MRI (top) shows an enlarged vein of Galen (arrowhead) arising from a large superior sagittal sinus (arrow) in the mutant; the coronal slice (middle) corresponds to the green line in the sagittal view and shows prominent vessels throughout the parenchyma. Midsagittal section of another mutant (bottom) shows a lesion in the olfactory bulb (arrow) arising from large anomalous veins (arrowhead). The normal caliber of the vein of Galen (arrow) is shown in the control. (**C**) Midsagittal 3D volumetric reconstruction of micro-CT (left) of the mutant from **B** (bottom) shows the lesion in the olfactory bulb (arrow) and the draining vessel (arrowhead). Coronal histologic section (right) of the same sample at original magnification ×10 stained with H&E shows a subarachnoid cerebrospinal fluid (CSF) cavity (arrow) between the olfactory bulbs surrounding the anterior portion of the falx, which has large veins in it (arrowhead), most likely consistent with CSF-venous fistula, as supported by the retrograde perfusion of polymer into this space seen on micro-CT. (**D**) Gross photograph of a suspected cavernous malformation (arrow) arising from a branch of the inferior vena cava (arrowhead) in the mutant. The right auricle (not visualized) is ligated. H, heart; L, lung. H&E-stained histologic section of the specimen reveals blood-filled dilated vascular channels associated with organizing thrombotic material and reactive inflammation consistent with cavernous angioma. Scale bar: 60 μm.

The syndromic mouse model showed vascular anomalies and malformations similar to those in the patients, including large dural sinuses (*n* = 3/5) (Figure 4A), large veins of Galen and veins throughout the parenchyma (*n* = 5/5) (Figure 4B), and malformations arising from these veins (*n* = 3/5) (Figure 4C), seen on micro-CT, MRI, gross dissection, and histology. Further, gross dissection in 1 specimen revealed a cavernous angioma off of a branch of the inferior vena cava (Figure 4D).

**Figure 5:**
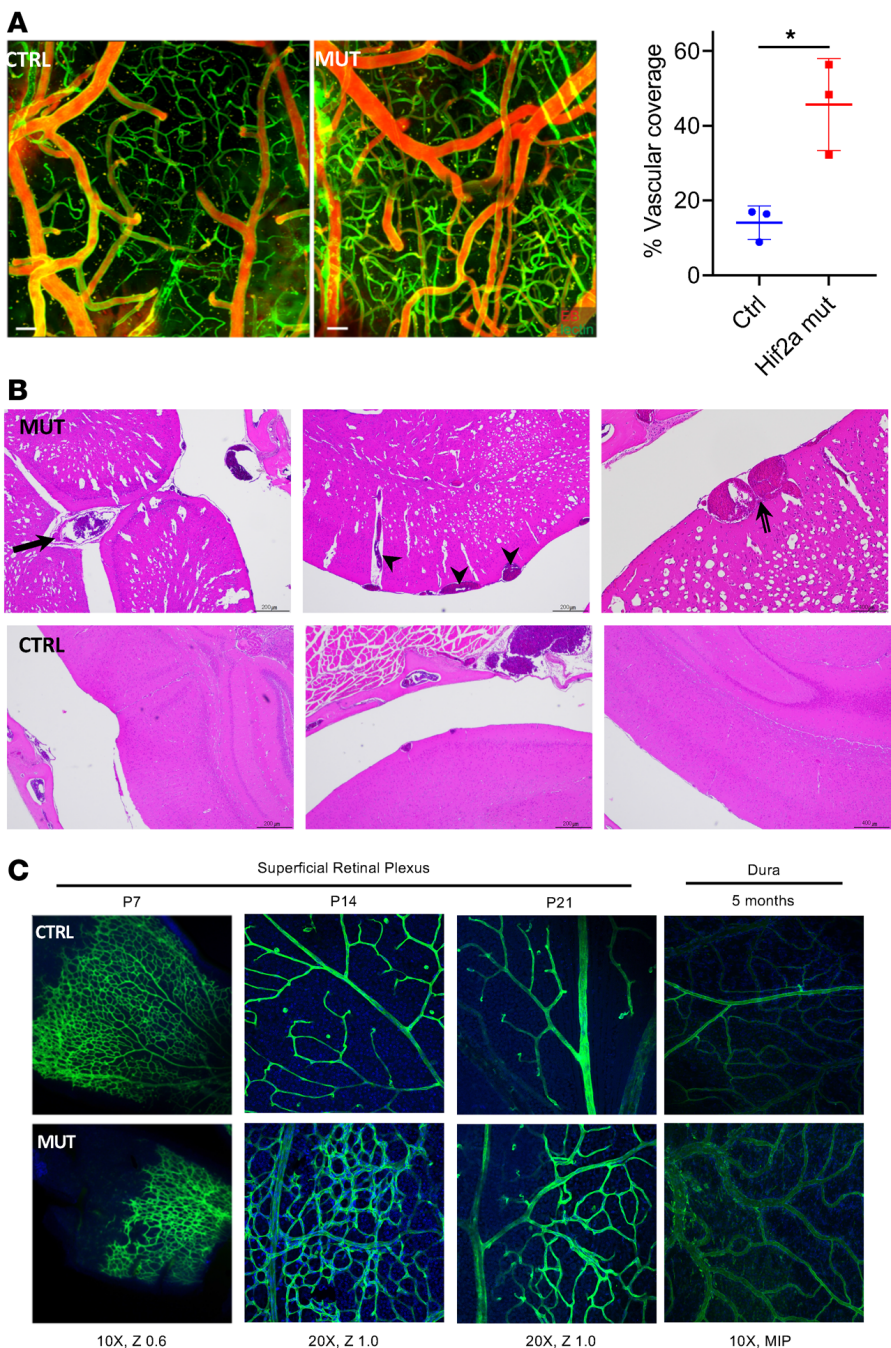
Failure of early vascular regression in *EPAS1* gain-of-function mouse model leads to persistent venous anomalies and malformations. (**A**) Representative images of intravital 2-photon microscopy of leptomeningeal and parenchymal vessels through a thinned skull window following retro-orbital i.v. injection for tomato lectin DyLight 488 and Evans blue demonstrated increased density and tortuosity of pial arterioles and venules and parenchymal capillaries in the mutant compared with the littermate control. Scale bar: 50 μm. Data represent the mean ± SEM. Quantification of *n* = 6 (3 mutant, 3 control) is shown; 2-tailed *t* test, *P* value equals 0.014. *P < 0.05. (**B**) H&E staining of the mouse model brain shows a developmentally large vein of Galen (arrow), large leptomeningeal and parenchymal veins (arrowheads), and cavernous angiomas arising directly from these veins (double-lined arrow); controls are shown. Large veins throughout enlarged Virchow-Robin spaces are also seen in the mutant. Scale bars: 200 μm (top left and top middle), 400 μm (top right); for the CTRL: 200 μm (bottom left and bottom middle), 400 μm (bottom right). (**C**) Representative images of isolectin B4 staining of the mouse model superficial plexus of the retina at postnatal time points 7, 14, and 21 days (P7–P21), during which the retinal vasculature of the mouse completes development, demonstrates that the plexiform network of veins fail to regress in the mutant compared with the control. This persists in the dura as evaluated at 5 months old.

Intravital 2-photon microscopy of leptomeningeal and parenchymal vessels through a thinned skull window in the transgenic mouse model, following retro-orbital intravenous injection of tomato lectin DyLight 488 and Evans blue, demonstrated increased density and tortuosity of pial arterioles and venules as well as parenchymal capillaries in the mutant compared with the littermate control (Figure 5A). Histology confirmed these findings and demonstrated postcapillary subarachnoid malformations consistent with cavernous malformations arising directly from large leptomeningeal veins (Figure 5B and Supplemental Figure 11). Staining of the superficial plexus of the retina during the corresponding developmental time points revealed a persistence of a plexiform embryonic venous network in the syndrome model compared with the control; this persists in the adult dura (Figure 5C); additional sample imaging and quantification can be found in Supplemental Figure 12 and Supplemental Figure 13.

## Discussion

We identified a spectrum of vascular malformations arising from multifocal irregular vascular development in the *EPAS1* gain-of-function syndrome patients as well as the transgenic mouse model. These included retinal capillary hemangiomas, ocular venous malformations resulting in congenital malformation of the optic disc, intracranial venous and cavernous malformations, and malformations of the extrinsic and intrinsic spinal venous systems resulting in dysraphism. We characterized the novel subfascial malformations on the posterior aspect of the cervical spine arising directly from these anomalous veins as cavernous malformations based on 2 characteristics. First, these lesions are not intraparenchymal and, therefore, do not fit the criteria of hemangioma (19, 20). Second, these lesions do not contrast enhance in the same phase as the arteries and veins, consistent with the angiographically occult nature of cavernous malformations (21, 22). These 9 patients currently range in age from 16 to 63 years old and have been followed at the NIH for up to 11 years (patient 1). None of the patients have developed nonocular neurologic signs due to these malformations; patient 7 had a clinically silent frontal lobe venous infarct, and patient 8 had retinal hemorrhages due to the central retinal venous malformation surrounding the macula. Although all patients with morning glory anomaly or significant optic disc malformation had poor visual acuity, this remained stable throughout their follow-up. The venous and cavernous malformations in syndromic patients appeared similar to sporadic malformations, but hemorrhage in syndromic patients was restricted to the cases previously described and myelopathy did not occur, suggesting that syndromic malformations are less prone to hemorrhage than sporadic ones (22, 23).

The pattern of anomalous veins throughout the spine appears consistent with remnant intersomitic or segmental veins, which would normally regress during development as arterial branches from the dorsal aorta develop (24). Early in development, a hypoxic gradient is established in the ventral-dorsal axis based on the implantation of the embryo (8, 25). The ventral aspect of the embryo is better oxygenated than the dorsal aspect (25). A sinusoidal and plexiform network of veins are the first vessels to organize within dorsal mesenchyme once the removal capacity of metabolic waste by passive diffusion is exceeded (26). These anterior, middle, and posterior plexuses are drained by lateral head veins, which will develop into the major dural sinuses and jugular veins (27).

A common developmental mechanism leading to vascular malformations in the syndrome would be failure of normal venous regression that occurs in development owing to increasing oxygen tension. This mechanism is supported by the co-occurrence of (a) the spinal venous malformations in persistent dorsal mesenchyme connecting to remnant lateral and segmental veins and draining to the inferior vena cava; (b) large superior sagittal sinus and vein of Galen confluence, which develop from the common central head vein; and (c) rete mirabile. Moreover, the large intracranial dural sinuses, anomalous posterior condylar emissary veins, and subfascial cavernous malformations drain laterally to large anomalous deep cervical veins. This is reminiscent of the embryonic drainage of the mesenchymal plexuses to lateral head veins, suggesting an arrested developmental configuration (27).

The transgenic mouse model for *EPAS1* gain-of-function syndrome demonstrated that this mutation is sufficient to cause similar anomalies and malformations to those observed in the patients. In addition, the model supported the previously stated hypothesis by demonstrating the same vascular malformations and by showing failure of venous regression in the eye and dura, which have a shared venous development (28).

Although these patients are known to have varying levels of mosaicism as previously reported, we observed similar anomalies in all patients (29). We believe that the gain-of-function mutation in *EPAS1* more significantly affects endothelial cells, where it is highly expressed in development (8). In fact, our measurement of allele frequency in tumor vessels confirmed a higher degree of mosaicism in vessels compared with nontumor adjacent normal tissue. In addition, the persistence of remnant dorsal mesenchyme along the spine and prominent leptomeninges, which are of mixed mesenchymal and neural crest origin, (30) surrounding the anomalous intracranial veins suggests a failure of neural crest epithelial-mesenchymal transition (18, 31). This may explain the nonparenchymal locations of some of the malformations.

These newly observed phenotypes together with those previously reported, including paraganglioma, polycythemia, and duodenal ampullary somatostatinoma (1, 2), suggest that the initial somatic mutation arises early in development in a precursor cell lineage that is capable of giving rise to angioblasts, mesenchymal cells, and neural crest. However, the variant allele frequency measured in the patient samples, which demonstrates that surrounding normal tissue has a much lower variant allele frequency than tumor vessels, suggests that there may be a cell- or tissue-specific process that selects for or against proliferation of cells with the *EPAS1* gain-of-function mutation. Additionally, the locations of these pathologies in the syndrome and the role of *EPAS1* in developmental epithelial-mesenchymal transition suggests a failure of migration of the affected cells into their proper locations.

Traditionally, cerebral cavernous malformations, which are often found with associated developmental venous anomalies, are intraparenchymal lesions (22, 32). They may occur in an autosomal dominant inherited or somatic fashion owing to mutation in the CCM1–3 genes (33, 34). Subarachnoid cavernous malformations, which we identified in several patients, have rarely been reported (35). Our findings support a common developmental mechanism for cavernous malformations and developmental venous malformations. This study suggests that the relationship between the spectrum of developmental vascular malformations and neural crest tumors found in these patients is mediated by HIF-2α.

This study adds another clinical feature to *EPAS1* gain-of-function syndrome and provides additional understanding of the role of HIF-2α in vascular development. We demonstrate that somatic gain-of-function mutation in *EPAS1* in early development prevents normal venous system regression and pruning, resulting in excessive venous elements. Constitutive activation of HIF-2α prevents the normal reduction in HIF-2α activity that occurs after vascular development increases tissue oxygen levels to normal (36). Additionally, this mechanism may apply to the development of sporadic cavernous and venous malformations that to date remain unexplained.

## Methods

### Patient selection and evaluation

Patients met the criteria of the syndrome, including paraganglioma, polycythemia, and confirmed *EPAS1* somatic mutation (Supplemental Table 1) (17, 28). Following the recent findings of posterior fossa and spine malformations in patients 1–8 and anomalies of the choroid and retina in patients 1, 2, and 4, 8 of 9 patients underwent ophthalmologic exam, which included fundoscopy and optical coherence tomography imaging, and all patients underwent further neurologic imaging evaluation (13, 17). Additional anatomic imaging for tumor staging and restaging included MRI and/or CT of head and neck and CT of the chest, abdomen, and pelvis. We evaluated available imaging for vascular malformations.

### Laboratory studies

#### Patient EPAS1 mutation analysis.

Mutations of the ODD of HIF-2α were confirmed in the genomic DNA extracted from either multiple tumor tissues and/or leukocytes in each patient (Supplemental Table 1) by either Sanger sequencing or peptide nucleic acid sequencing as previously described (1, 28). Although Sanger sequencing was initially used to detect the mutations in the tumor tissue (1), it was unable to detect the mutation in circulating leukocytes; these mutations were later detected in these patients by either peptide nucleic acid sequencing or ddPCR, which we used here to determine variant allele frequency.

#### Transgenic knockin mouse model.

*Epas1*^A529V^ mutant mice were established as previously described via TALEN-mediated homologous recombination (36). A point mutation, A529V (GCA>GTA), corresponding to the mutation detected in the index patient, was introduced in exon 12 of *Epas1* in 1 allele distal to an inserted reverse-oriented neomycin resistance gene cassette flanked by *loxP* sites in intron 11, which in its native state is expected to inhibit transcription of the altered *Epas1* allele (1, 28). This heterozygous mouse (*Epas1*^+/Neo^*-*^A529V^) does not demonstrate polycythemia or noradrenergic phenotype (36). Crossing it with a transgenic mouse of Cre recombinase under the control of the adenovirus Enzyme IIa cyclization recombinase promoter (EIIa-Cre), which is expressed in most tissues but not all cells in the early postzygotic embryo, leads to sporadic excision of the *loxP* flanking neomycin resistance gene cassette in all tissues (37). This yields 4 potential categories of mice from each litter: (a) *Epas1*^+/Neo^*-*^A529V^ EIIa-Cre, referred to as mutant; (b) *Epas1*^+/+^ EIIa-Cre, referred to as the Cre-only control; (c) the *Epas1*^+/Neo^*-*^A529V^, referred to as heterozygous mouse; and (d) *Epas1*^+/+^, referred to as WT control (variant negative, Cre negative) (17). This strategy results in the *EPAS1* gain-of-function mosaic mutant mouse used in our study (*Epas1*^+/Neo^*-*^A529V^ EIIa-Cre) (37, 38). WT or Cre-only mice were used as controls for all experiments. Previous quantification of this allele using ddPCR in reverse-transcribed cDNA in multiple tissues showed Cre-mediated excision occurred in a high percentage of cells in all tissue types tested (36).

Experiments were performed on 3- to 5-month-old mice for all experiments except intravital 2-photon microscopy, for which 8-month-old mice were used (1 mutant, 1 Cre-only control), and retinal development analysis via immunofluorescent staining and confocal imaging, for which postnatal day 7, 14, and 21 mice were used (2 mutant, 2 Cre-only control for each time point). Five mutant mice (4 males, 1 female) and 4 control mice (2 males, 2 females) underwent Microfil vascular perfusion and subsequent ex-vivo 14T MRI, micro-CT scans and volumetric reconstruction, and gross dissection.

#### Intravital 2-photon microscopy of pial and parenchymal vessels.

The surgical procedure for intravital 2-photon microscopy was performed per protocol and based on adapted techniques previously developed (39). Three mice — one 8-month-old mutant and littermate control and two 15-month-old mutant and littermate control pairs, all female — were anesthetized using ketamine, xylazine, and acepromazine. The hair over the scalp was removed using clippers. Lidocaine was applied to the scalp, which was then cleaned with ethanol. Subsequently, a scalp incision was made, the skull was exposed, and a metal bracket was affixed to the skull with glue, leaving an exposed circular area of the skull. A 2 × 2 mm cranial window of 10–15 μm thickness was fashioned using a high-speed microdrill (39).

For blood vessel visualization, 100 μL 0.1 mg/mL Evans blue (MilliporeSigma) and 100 μL tomato lectin DyLight 488 (Vector Laboratories) was injected i.v. via the retro-orbital sinus prior to imaging. Mice were then imaged through the cranial window using a Leica SP8 2-photon microscope with an 8000 Hz resonant scanner, a 25× collar-corrected water-dipping objective (1.0 NA), a quad HyD external detector array, a Mai Tai HP DeepSee Laser (Spectra-Physics) tuned to 905 nm (for GFP and DyLight 488), and an Insight DS laser (Spectra-Physics) tuned to 1050 nm (for Evans blue). The 3D images were captured using *Z*-stacks of 100 μm in depth (2.5 μm step size). The 3D image stacks obtained were subsequently imported to Imaris version 9.0 software (Bitplane) for further analysis and to generate maximal projections.

Following intravital imaging, images were imported to Imaris 9.0. The volume of vascular coverage was quantified using the surfaces function to mask Evans blue–positive and tomato lectin Dylight 488–positive vessels and calculate their volume. The total vessel volume was then reported as a fraction of the total volume of the volume of the 3D image acquired.

#### Vascular perfusion of mouse model.

Following heparinization, carbon dioxide narcosis, and bilateral thoracotomy, the vasculature of the mouse was casted with Microfil silicate polymer (Flow Tech, Inc.), mixed in a 2:7:1 (Microfil:diluent:polymerizing agent) ratio optimized to allow arterial-venous transit, through a 30G needle secured in the exposed thoracic aorta as previously described (17). This was modified from a previous method (40). Direct visualization of complete filling of the diploic veins was used as the endpoint (17).

#### Ex vivo imaging of mouse model head and spine.

Intact polymer-casted mouse samples were scanned via 14T MRI (Bruker BioSpin) and Bruker SkyScan1172 Micro-CT (Micro Photonics, Inc.), as previously described (17). Horos Imaging Software and Bruker software suite for MRI and micro-CT imaging facilitated image reconstruction and analysis, respectively (17).

#### Histology.

For whole head histology and microscopy, FFPE tissue specimens from mice were cut in 4 μm sections and then transferred onto charged glass slides. After deparaffinization in xylene and subsequent rehydration in a series of graded ethanol baths, specimens were either stained using H&E or underwent immunohistochemistry on a Leica Bond Max automated staining system. Anti-CD34 (Abcam ab185732) was probed using citrate antigen retrieval. Histologic slides were viewed using a Nikon Eclipse Ci microscope. Images were digitally stored at original magnification ×2 and ×4 using Nikon DS-L4 software.

#### Immunofluorescence and confocal imaging.

The superficial vascular plexus of the retina was evaluated via whole mount, immunofluorescent staining, and confocal imaging in a total of 12 mice: 2 mutant and 2 littermate Cre-only control mice at each postnatal day 7, 14, and 21. The eyes were enucleated. The optic nerve, extraocular muscles, and connective tissues were removed posteriorly, and the cornea and lens were removed anteriorly. The retina was removed intact and then divided into quadrants for flat whole mount on a charged glass slide.

The dura of the calvarium was evaluated intact. A midline incision was made in the scalp to expose the calvarium from the frontonasal suture to the cranio-cervical junction. The dorsal cranio-cervical muscles were elevated from the skull base, and the temporalis muscles were elevated from the temporal fossa. The calvarium and dura were circumferentially elevated with the initial incisions made through the external auditory canal bilaterally; this was connected anteriorly to the frontonasal suture and posteriorly to the lambdoidal suture. For confocal imaging, the calvarium was placed in a 17 mm glass bottom WillCo-dish (WillCo Wells) with the dura facing up.

Both retinal whole mount slides and calvaria samples underwent the same staining process as follows. Samples were washed in 1× PBS with 0.2% Triton-X (T-PBS) and subsequently blocked with 5% BSA in T-PBS overnight at 4°C. Samples were then incubated at room temperature in 0.5% T-PBS for 5 minutes, 3 times, and then in 0.1 mM CaCl_2_, 20 μg/μL isolectin GS-IB_4_–Alexa Fluor 488 antibody (Invitrogen, Thermo Fisher Scientific, I21411) in 5% BSA in T-PBS overnight at 4°C. Samples were washed in T-PBS and incubated overnight at 4°C. Samples were then incubated in DAPI (1:1000) overnight at 4°C. Samples were then washed in 0.5% T-PBS for 4 hours followed by 0.1 mM CaCl_2_/PBS at room temperature. All confocal imaging was performed on the Zeiss LSM 780. For the dural samples, the objective lens was EC Plan-Neofluar 10×/0.3, the pixel size was 0.83 μm, and the optical section was 1.9 μm.

Retinal confocal images were evaluated using AngioTool to quantify junction density, lacunarity, and vessel percentage per unit area (41).

#### Manual microdissection and determination of allele frequency.

Variant *EPAS1* allele frequency was measured in resected paraganglioma/pheochromocytoma in 2 patients. Tumor, adjacent vessel, and adjacent nontumor/nonvessel tissues were manually microdissected from paraffin-embedded sections. Genomic DNA was extracted from these microdissected samples and used for ddPCR, which was performed with the Bio-Rad QX200 ddPCR system in the Genomics Core facility of the NCI Center for Cancer Research, according to the manufacturer’s instructions. Variant allele frequency measurement assay was designed based on the human *EPAS1* genomic sequence with the Bio-Rad website tool. The probe for the WT allele was labeled with hexachloro fluorescence, and the probe for the *EPAS1* mutant allele (P531S) was labeled with fluorescein fluorescence. The results were analyzed with the QuantaSoft software (Bio-Rad).

### Statistics

Data were analyzed via 2-tailed unpaired *t* test when appropriate. A *P* value of less than 0.05 was considered significant.

### Study approval

The IRB of the *Eunice Kennedy Shriver* National Institute of Child Health and Development (NICHD, ClinicalTrials.gov identifier NCT00004847) approved the study protocol. Written informed consent was obtained from all participants. The research study followed all applicable institutional and governmental regulations concerning the ethical use of animals (NICHD animal study proposal 18-028).

## Author contributions

JSR conceived the study, identified the malformations, performed experiments, collected and analyzed data, and drafted and revised the manuscript. HW generated the transgenic mouse model, performed allele frequency experiments, and revised the manuscript. PMD performed retinal whole mount staining and confocal imaging and reviewed the manuscript. AJC collected mouse MRI, micro-CT, and dural confocal data; evaluated imaging data; quantified retinal confocal data; and drafted and revised the manuscript. PM performed intravital 2-photon microscopy mouse experiments, analyzed data, and revised the manuscript. CX performed allele frequency experiments. AJ collected and analyzed patient data and revised the manuscript. NE performed histology and staining and revised the manuscript. DRD and JM performed ex vivo imaging experiments and revised the manuscript. MAN collected and analyzed patient data and revised the manuscript. RHK performed vascular perfusion mouse experiments, collected and analyzed data, and revised the manuscript. BRR analyzed patient data and drafted and revised the manuscript. JGS, AP, and RFS analyzed patient data and revised the manuscript. AV analyzed patient and mouse experimental data and revised the manuscript. MRG analyzed patient data and revised the manuscript. DBM analyzed mouse experimental data and revised the manuscript. EC collected and analyzed patient ophthalmologic data and revised the manuscript. BAK and JDH analyzed patient and mouse data and revised the manuscript. ZZ and KP conceived the study, analyzed patient and mouse data, and revised the manuscript.

## Supplementary Material

Supplemental data

## Figures and Tables

**Table 1:**
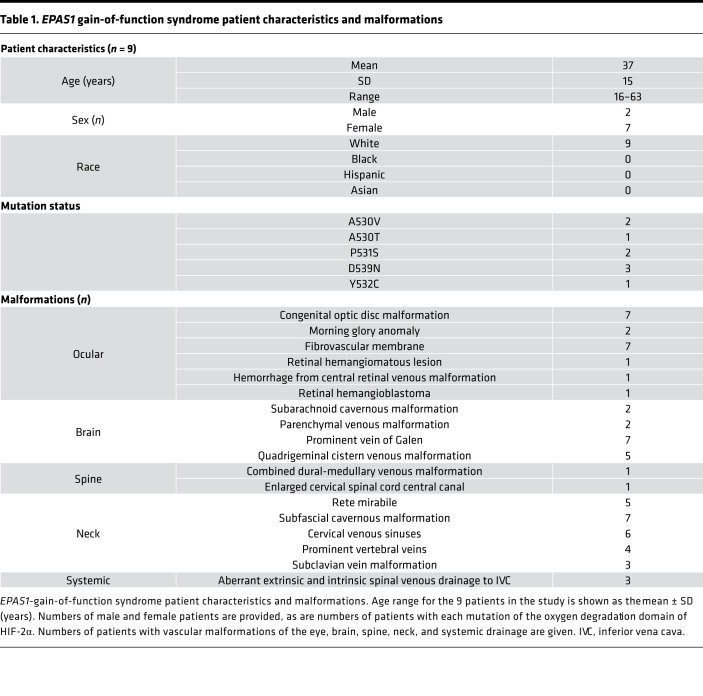
*EPAS1* gain-of-function syndrome patient characteristics and malformations

## Version 1 01/26/2021

In-Press Preview

## Version 2 03/08/2021

Electronic publication
