# Supplementary material for: Developmental vascular malformations in EPAS1 gain-of-function syndrome
Source: JCI Insight. 2021 Mar 8;6(5):e144368. doi: 10.1172/jci.insight.144368 (PMC8021124; doi:10.1172/jci.insight.144368)
Supplement: Supplemental data [file jciinsight-6-144368-s298.pdf]

ONLINE-ONLY MATERIALS

Online-only Tables

Supplemental Table 1. Characteristics of *EPAS1*-Associated Syndrome Patients									
Patient	1	2	3	4	5	6	7	8	9
Sex	F	M	F	F	F	M	F	F	F
Age at Present	38	21	16	30	52	33	46	63	36
Age at Onset of Diagnosed Condition (years)									
Polycythemia	Birth	2	7	Birth	7	2.3	2	17	31
PPGL	14	15	8	18	35	16	39	56	31
Ampullary Somatostatinoma	29	17	-	22	35	-	39	-	-
Mutation Analysis—Tumors and Circulating Leukocytes									
EPAS1	A530T	P531S	D539N	A530V	Y532C	D539N	A530V	D539N	P531S
Prothrombin	Negative	Negative	Negative	Negative	Negative	Negative	Negative	Negative	G20210A
EPOR, HIF-1 α, JAK2, PHD1/2, SDHB/C/D, VHL	Negative	Negative	Negative	Negative	Negative	Negative	Negative	Negative	Negative
Clinical Characteristics at Presentation at NIH									
Blood Pressure (mm Hg)	113/78	126/76	125/59	135/78	111/69	141/85	89/49	140/55	136/86
Heart Rate (bpm)	88	82	98	105	71	75	137	83	75
Pertinent Family History									
	None	None	None	None	None	None	None	None	None
Laboratory Values at Presentation at NIH									
Complete Blood Count									
Erythrocytes (per mm^3)	7.49 (3.93-5.22)	6.24 (3.93-5.22)	6.38 (3.9-4.96)	7.85 (3.93-5.22)	4.94 (3.93-5.22)	8.01 (4.63-6.08)	6.85 (3.93-5.22)	5.42 (3.93-5.22)	5.85 (3.93-5.22)
Hematocrit (%)	48.5 (34.1-44.9)	50.7 (40.1-51.0)	45.9 (32.4-39.5)	59.3 (40.1-50.1)	32.6 (31.28-43.2)	51.1 (40.1-51.0)	49.3 (34.1-44.9)	38.5 (34.1-44.9)	51.3 (34.1-44.9)
Hemoglobin (g/dL)	16.2 (12.0-15.5)	16.2 (13.7-17.5)	14.5 (10.6-13.2)	18.8 (13.7-17.5)	9.2 (11.1-15.0)	15.3 (13.7-17.5)	15.4 (11.2-15.7)	11.3 (112-15.7)	17.9 (11.2-15.7)
Leukocytes (per mm^3)	7.98 (3.98-10.04)	8.55 (3.98-10.04)	4.10 (4.27-11.4)	8.13 (3.98-10.04)	3.54 (3.4-9.60)	4.93 (4.23-9.07)	5.64 (3.98-10.04)	8.17 (3.98-10.04)	5.45 (3.98-10.04)
Platelets (K/uL)	187 (173-369)	207 (173-369)	259 (199-367)	249 (173-369)	214 (162-380)	212 (161-347)	258 (173-369)	533 (173-369)	226 (173-369)
Erythropoietin (mIU/mL)	150 (3.7-31.5)	25.8 (2.6-18.5)	111 (3.7-31.5)	230 (3.7-31.5)	165 (3.7-31.5)	88.9 (2.6-18.5)	50.4 (3.7-31.5)	140 (2.0-18.5)	15.2 (2.6-18.5)
RDW (11.7-14.4%)	24.9	16.8	19	20.4	17.1	22.4	21.4	19.4	14.3
MCV (fL)	64.8 (79.8-94.8)	81.3 (79.0-92.2)	71.9 (75.9-87.6)	75.5 (79.4-94.8)	66.0 (55.0-99.0)	63.8 (79-92.2)	72 (79.4-94.8)	71 (79.4-94.8)	87.7 (79.4-94.8)
Plasma Biochemistry									
Normetanephrine (18-112 pg/mL)	4834	705	816	858	515	257	1993	787	237
Metanephrine (12-61 pg/mL)	121	53	72	9	22	52	77	47	18
Norepinephrine (pg/mL)	10951 (80-498)	921 (84-794)	1434 (112-750)	1760 (80-498)	775 (80-498)	348 (84-794)	1875 (112-750)	626 (112-750)	317 (112-750)
Epinephrine (pg/mL)	100 (4-83)	28 (0-57)	25 (<50)	7 (4-83)	9 (4-83)	<20 (<57)	24 (<50)	<20 (<50)	<20 (<50)
Chromogranin A (ng/mL)	1640 (<225)	130 (<93)	133 (<93)	320 (<225)	72 (0-51)	64 (<93)	114 (<93)	192 (<93)	106 (<93)

eTable 1. Characteristics of *EPAS1*-Associated Syndrome Patients. Sex, age at present and diagnosis of each component of the syndrome, mutation analysis, clinical characteristics, and laboratory values are shown for each patient with the syndrome. Note: Patient 3, confirmed for polycythemia at age 7, was suspected for polycythemia from birth due to her appearance. Abbreviations: endothelial PAS domain-containing protein 1, *EPAS1*; pheochromocytoma and paraganglioma, PPGL; erythropoietin receptor, EPOR; hypoxia-inducible factor-1 alpha, HIF-1α; janus kinase 2, JAK2; prolyl hydroxylase domain-containing protein 1/2, PHD1/2; succinate dehydrogenase subunits B/C/D, SDHB/C/D; von Hippel-Lindau, VHL; millimeter of mercury, mm HG; beats per minute, bpm; millimeters cubed, mm^3; grams per deciliter, g/dl; 103 per microliter, K/uL; milli-international unit per milliliter, mIU/mL; femtoliters, fL; picograms per milliliter, pg/mL; nanograms per milliliter, ng

Online-only Figures

eFigure 1.

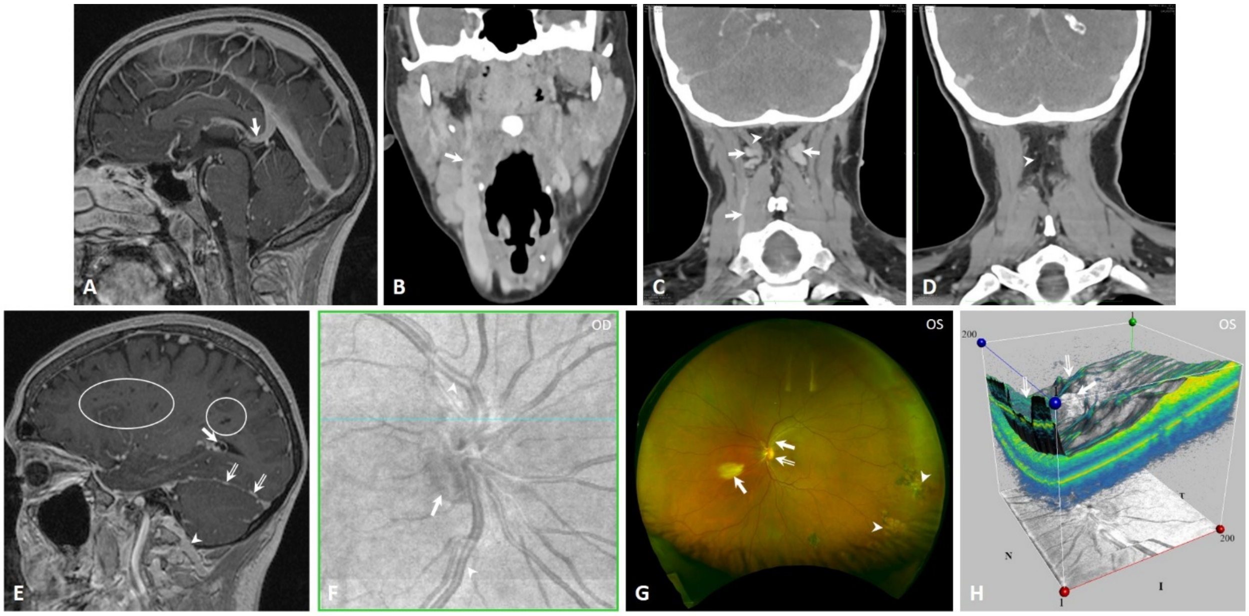

Supplemental Images Patient 1. *Panel A:* Sagittal T1-weighted post-contrast MRI of the head shows large superior sagittal sinus, straight sinus, and vein of Galen confluens (arrow). *Panel B:* Coronal CT of the neck with contrast shows rete mirabile of the right internal jugular vein and internal carotid artery (arrow). *Panel C:* Coronal CT of the neck with contrast shows the sub-fascial cavernous malformation (arrowhead) and venous drainage (arrows). *Panel D:* Coronal CT of the neck with contrast shows the same malformation (arrowhead). *Panel E:* Sagittal T1-weighted post-contrast MRI of the head shows prominent choroid plexus (arrow), prominent tentorial veins (double-lined arrows), and the large posterior condylar emissary vein (arrowhead). Prominent spaces with and without visualized veins within them are seen throughout the parenchyma (white circles); those without veins seen follow the orientation of the veins and resemble perivenular Virchow-Robins spaces. *Panel F:* Red free light fundoscopy of the right eye (OD) with 20/25 acuity shows a fibrovascular membrane (arrow) and large retinal veins (arrowheads). *Panel G:* Fundoscopy of the left eye (OS) with 20/25 acuity shows optic disc elevation without appreciation of the optic cup, suggestive of congenital optic disc anomaly (double-lined arrow), fibrovascular membranes (arrows), and peripheral hemangiomatous lesions (arrowheads), which are present in the other eye also (not shown). *Panel H:* 3D Visualization of optical coherence tomography of the left eye shows optic disc elevation (arrow); the fibrovascular membrane originating from the optic disc continuing along the vessels is seen (double-lined arrows).

eFigure 2.

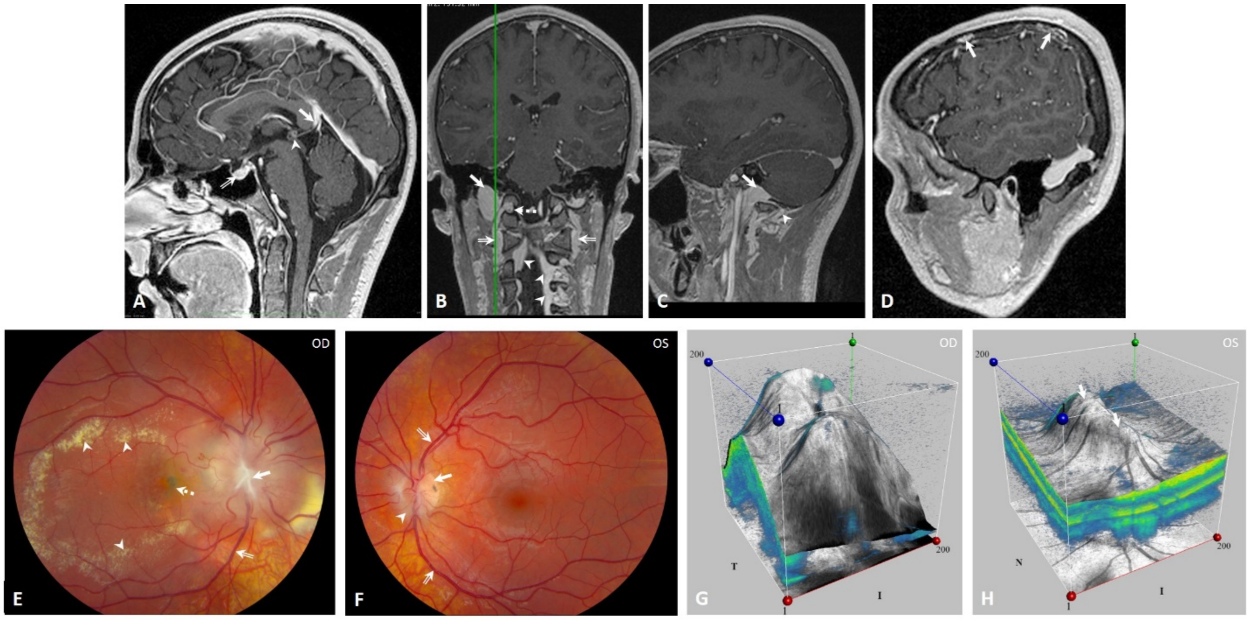

Supplemental Images Patient 2. *Panel A:* Sagittal T1-weighted post-contrast MRI shows a large superior sagittal sinus and vein of Galen confluens (arrow); venous malformation of the pineal gland is also seen (arrowhead). The pituitary is also mildly hypervascular (double-lined arrow). *Panel B:* Coronal T1-weighted post-contrast MRI shows a large jugular bulb (arrow) and prominent venous plexus at the cranio-cervical junction (double-lined arrow) arising from the posterior condylar emissary vein (dashed arrow) and draining to the cervical venous sinuses (arrowheads). *Panel C:* Sagittal plane corresponding to the green line in *Panel B* shows the jugular bulb (arrow) and the drainage of the posterior condylar emissary vein to the plexus (arrowhead). *Panel D:* Sagittal T1-weighted post-contrast MRI shows contrast enhancing lesions, likely vascular malformations of the bone, in the left calvarium (arrows). *Panel E:* Fundoscopy of the right eye (OD) with 20/400 acuity shows morning glory anomaly (arrow), large tortuous veins (double-lined arrow) with leakage, abnormality of the macula (dashed arrow) with edema and exudates and lipid accumulation (arrowheads). The retina is hypo- and hyper- pigmented throughout. *Panel F:* Fundoscopy of the left eye (OS) with 20/20 acuity shows optic disc elevation (arrow) and fibrovascular membrane originating along the vessels (arrowhead); the veins are large (double-lined arrows). *Panel G:* 3D Visualization of optical coherence tomography of the right eye shows the morning glory. *Panel H:* 3D Visualization of optical coherence tomography of the left eye shows optic disc elevation without optic cup (arrows).

eFigure 3.

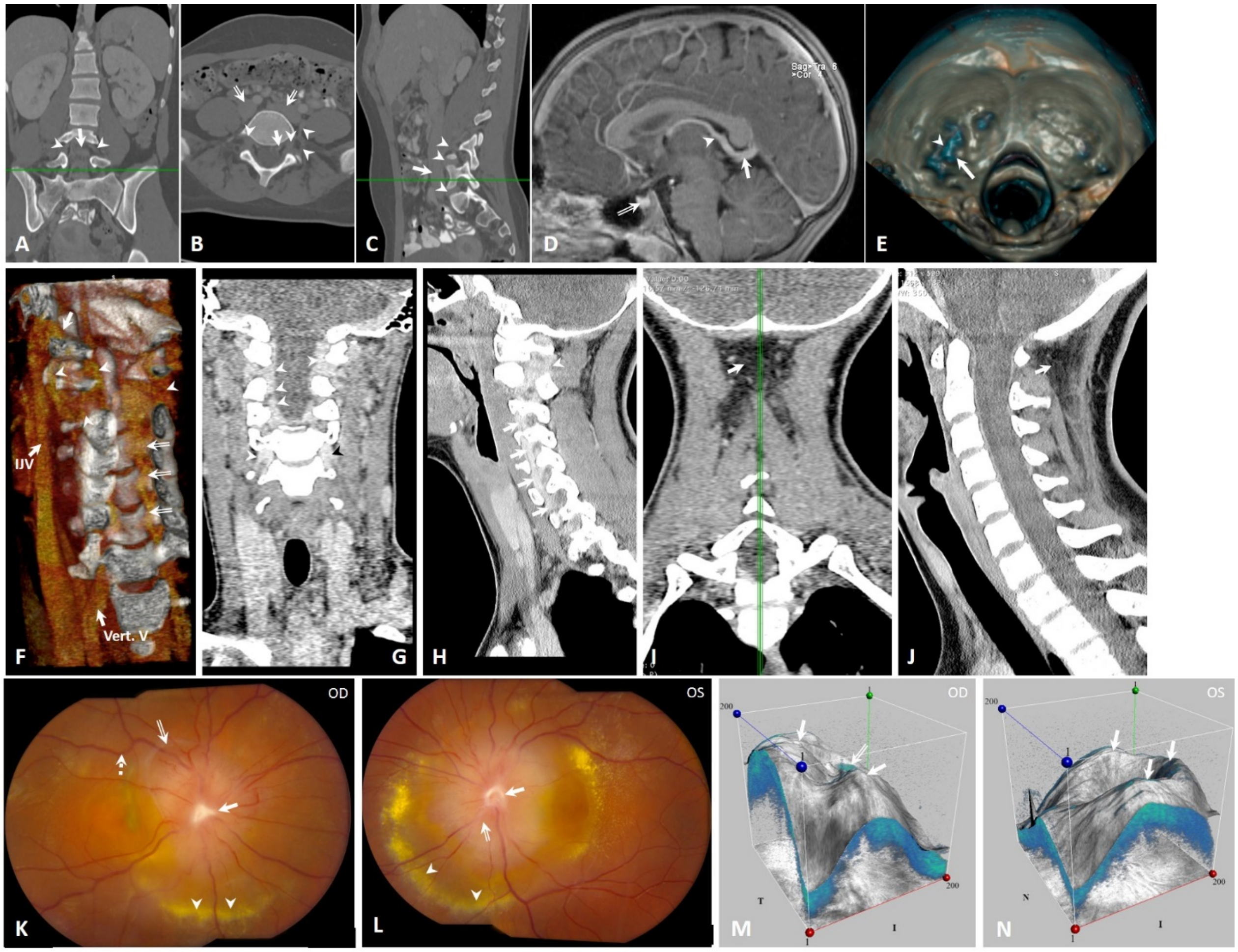

Supplemental Images Patient 3. *Panel A:* Coronal CT of the abdomen and pelvis with contrast shows a prominent epidural lumbar venous plexus (arrow) with drainage through plexiform intersegmental veins (arrowheads). *Panel B:* Axial CT of the abdomen and pelvis with contrast corresponding to the green line in *Panel A* shows the prominent epidural veins (arrow) draining to the intersegmental plexus bilaterally (arrowheads); these drain through the lumbar veins to the iliac veins (double-lined arrows). *Panel C:* Sagittal CT of the abdomen and pelvis shows the aberrant large plexiform lumbar veins (arrowheads) draining the prominent epidural veins to the inferior vena cava (arrow). *Panel D:* Sagittal T1-weighted post-contrast MRI shows a large confluens of the vein of Galen (arrow) and internal cerebral vein (arrowhead). The pituitary appears mildly hyper-vascular (double-lined arrow). *Panel E:* 3D volumetric reconstruction of CT of the head with contrast shows a defect in ossification of the right occipital bone (arrow); a vein (arrowhead) is seen in the uncalcified cartilage (blue). *Panel F:* 3D volumetric reconstruction shows the cranio-cervical venous plexus (arrowheads) arising from the internal jugular vein (IJV) and draining into the cervical venous sinuses and segmental veins throughout the cervical spine (double-lined arrows). This drains to the vertebral vein (Vert. V). *Panel G:* Source image for *Panel F* from coronal CT of the neck with contrast shows the cervical venous sinuses and segmental veins bilaterally (arrowheads). *Panel H:* Source image for *Panel F* from sagittal CT of the neck with contrast shows the cranio-cervical venous plexus (arrowhead) and the foraminal plexus drainage at each level (arrows). *Panels I-J:* Coronal and Sagittal CT of the neck with contrast, respectively, show the subfascial cavernous malformation on the posterior aspect of the neck (arrows). *Panel K:* Fundoscopy of the right eye (OD) with 20/50 acuity shows morning glory anomaly (arrow) with fibrovascular membrane (double-line arrow) originating from its center; lipid accumulation surrounding macular edema is seen (arrowheads). The veins are large and tortuous (dashed arrow). *Panel L:* Fundoscopy of the left eye (OS) with 20/100 acuity shows optic disc anomaly (arrow) with a fibrovascular membrane originating from the central vessels (double-lined arrow); lipid accumulation surrounding

disc edema is seen (arrowheads). *Panel M:* 3D Visualization of optical coherence tomography of the right eye shows the morning glory anomaly with optic disc elevation (arrows) and fibrovascular membrane along the vessels (double-lined arrow). *Panel N:* 3D visualization of optical coherence tomography of the left eye shows the optic disc anomaly with optic disc elevation (arrows).

eFigure 4.

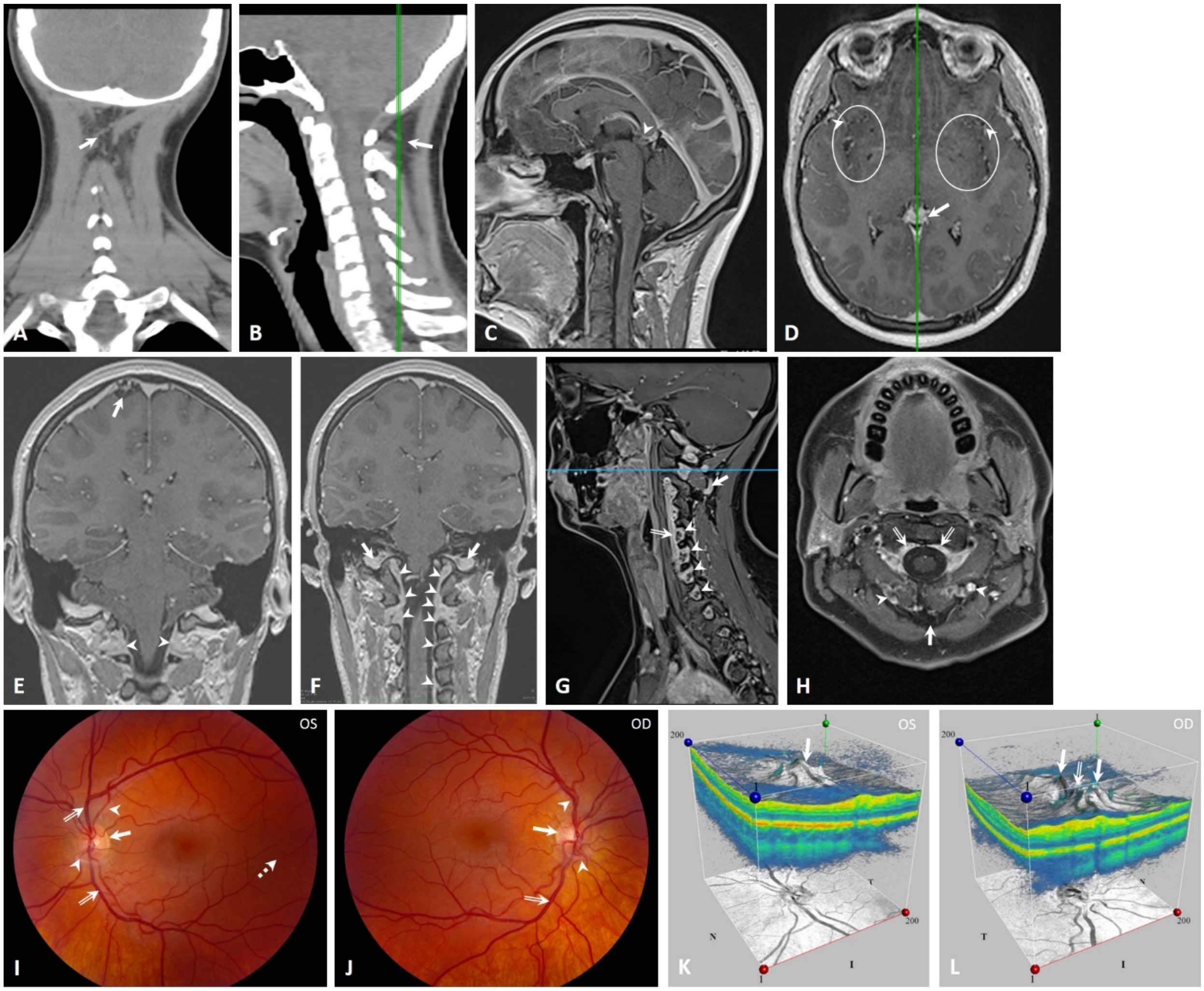

Supplemental Images Patient 4. *Panel A:* Coronal CT of the neck shows the subfascial cavernous malformation with draining veins (arrow); *Panel B* shows the corresponding sagittal slice. *Panel C:* Sagittal T1-weighted post-contrast MRI shows the prominent superior sagittal sinus and straight sinus; aberrant venous drainage of the pineal gland and tectum, to the vein of Galen confluens is seen (arrowhead). *Panel D:* Axial slice corresponding to the sagittal in *Panel C* (green line) shows the broad confluens of the vein of Galen (arrow); prominent Virchow-Robins spaces with venous plexuses (circles) are seen draining to the Sylvian veins (arrowheads). *Panel E:* Coronal T1-weighted post-contrast MRI of the head shows a suspected subarachnoid cavernous malformation with venous drainage to the superior sagittal sinus (arrow). Venous drainage of the posterior condylar emissary vein through an aberrant dural sinus at the cranio-cervical junction is seen bilaterally (arrowheads). *Panel F:* Coronal T1-weighted post-contrast MRI shows the cranio-cervical venous sinus continuing throughout the upper cervical spine (arrowheads); the jugular bulbs are seen (arrows). *Panel G:* Sagittal T1-weighted DIXON MRI sequence shows the venous drainage of the sub-fascial cavernous malformation at the lateral skull base (arrow). Prominent foraminal venous plexuses (arrowheads) at each level are seen draining into a prominent vertebral vein (double-lined arrow). *Panel H:* Corresponding axial slice (blue line in *Panel G*) shows dysraphism due to the neck malformation (arrow) which drains to large veins of the external system (arrowhead); these drain into the prominent extradural veins and dural sinuses (double-lined arrows). *Panel I:* Fundoscopy of the left eye (OS) with 20/16 acuity shows optic disc elevation (arrow), fibrovascular membrane (arrowheads) along the vessels originating from the optic disc; the veins are large (double-lined arrows) and the arteries are tortuous. Small retinal hemorrhages are present in the nasal periphery (dashed arrow) *Panel J:* Fundoscopy of the right eye (OD) with 20/16 acuity shows optic disc elevation and anomaly (arrow), fibrovascular membrane

(arrowheads), and large veins (double-lined arrow) with tortuous arteries. *Panel K:* 3D visualization of optical coherence tomography of the left eye shows optic disc elevation (arrow). *Panel L:* 3D visualization of optical coherence tomography of the right eye shows optic disc elevation (arrows) surrounding the optic disc anomaly (double-lined arrow).

eFigure 5.

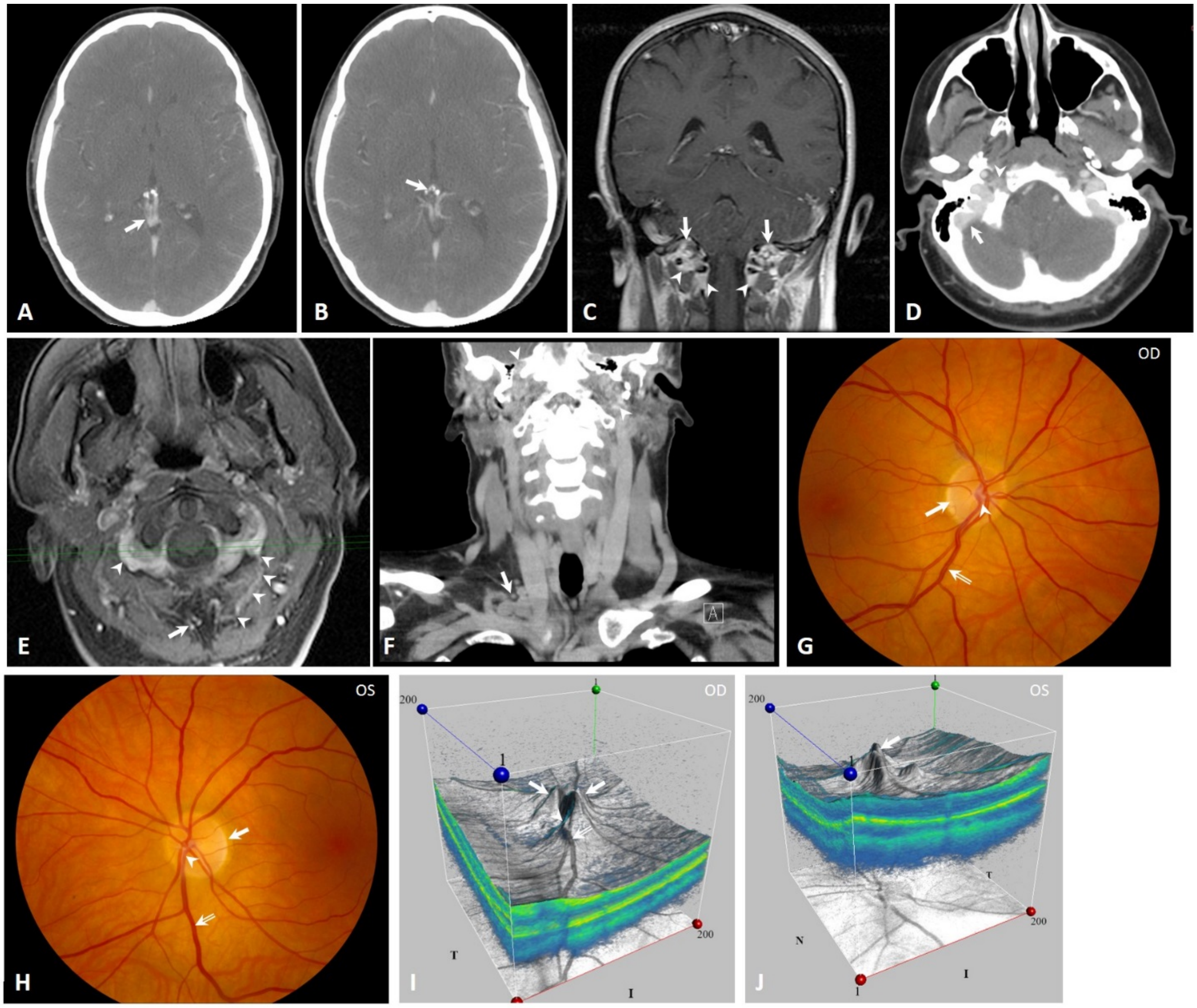

Supplemental Images Patient 5. *Panel A:* Axial CT of the head with contrast shows a prominent confluens of the vein of Galen (arrow); ventricular asymmetry is also noted. *Panel B:* Axial CT of the head with contrast one slice below shows a venous malformation (arrow) within the quadrigeminal cistern draining to the confluens of the vein of Galen. *Panel C:* Coronal T1-weighted post-contrast MRI sequence shows drainage of the posterior condylar emissary vein (arrows) to a prominent cranio-cervical venous plexus (arrowheads). *Panel D:* Axial CT of the head with contrast shows abnormal development of the right sigmoid sinus and petrous bone (arrow) with a plexiform venous malformation of the right petroclival joint draining to the jugular bulb (arrowhead) and surrounding the carotid artery. *Panel E:* Axial T1-weighted post-contrast MRI sequence corresponding to Panel A shows the sub-fascial cavernous malformation and dysraphism on the posterior aspect of the neck (arrow) draining through large veins (arrowheads) to the venous sinuses and plexus of the cranio-cervical junction. *Panel F:* Coronal CT of the neck with contrast shows the same venous plexus exiting the skull base (arrowheads) surrounding the jugular and carotid, likely a rete mirabile. A venous malformation arising from the right subclavian vein is also seen (arrow). *Panel G:* Fundoscopy of the right eye (OD) with 20/20 acuity shows optic disc elevation without optic cup (arrow); a fibrovascular membrane (arrowhead) is present at the center of the optic disc. The veins are large (double-lined arrow). *Panel H:* Fundoscopy of the left eye (OS) with 20/16 acuity shows optic disc elevation without optic cup (arrow); a fibrovascular membrane is present centrally (arrowhead). The veins are large (double-lined arrow). *Panel I:* 3D visualization of optical coherence tomography of the right eye (OD) shows optic disc elevation (arrows) with a fibrovascular membrane

(arrowhead) overlying a central vessel (double-lined arrow). *Panel J:* 3D visualization of optical coherence tomography of the left eye (OS) shows optic disc elevation (arrow).

eFigure 6.

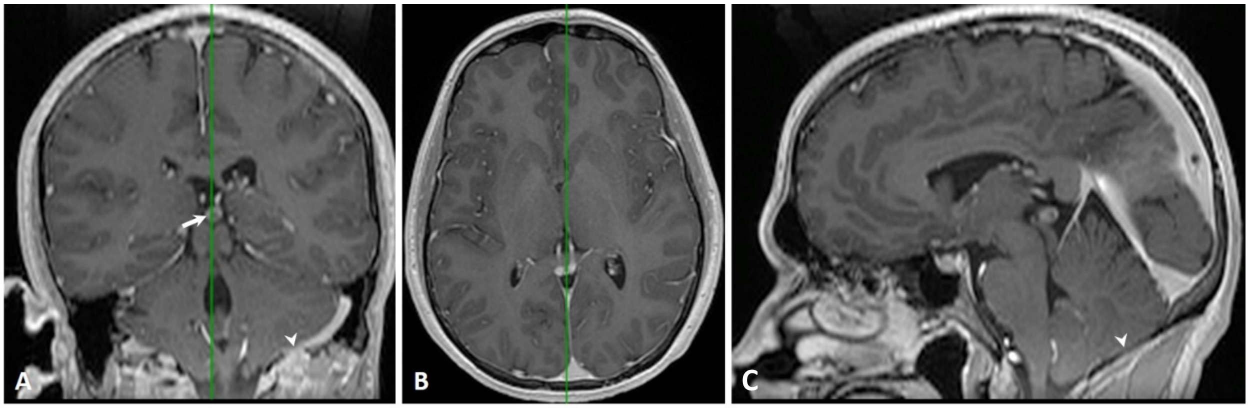

Supplemental Images Patient 6. *Panel A:* Coronal T1-weighted post-contrast MRI shows a venous malformation (arrow) connected to the choroid plexus through the velum interpositum. The left occipital bone is disrupted by a large plexiform posterior condylar emissary vein (arrowhead) draining the sigmoid sinus to the cranio-cervical plexus. *Panel B:* Corresponding axial slice. *Panel C:* Corresponding sagittal slice shows the same malformation (arrow); the right cerebellar tonsil is below the foramen magnum and the supraoccipital bone is narrow (arrowhead).

eFigure 7.

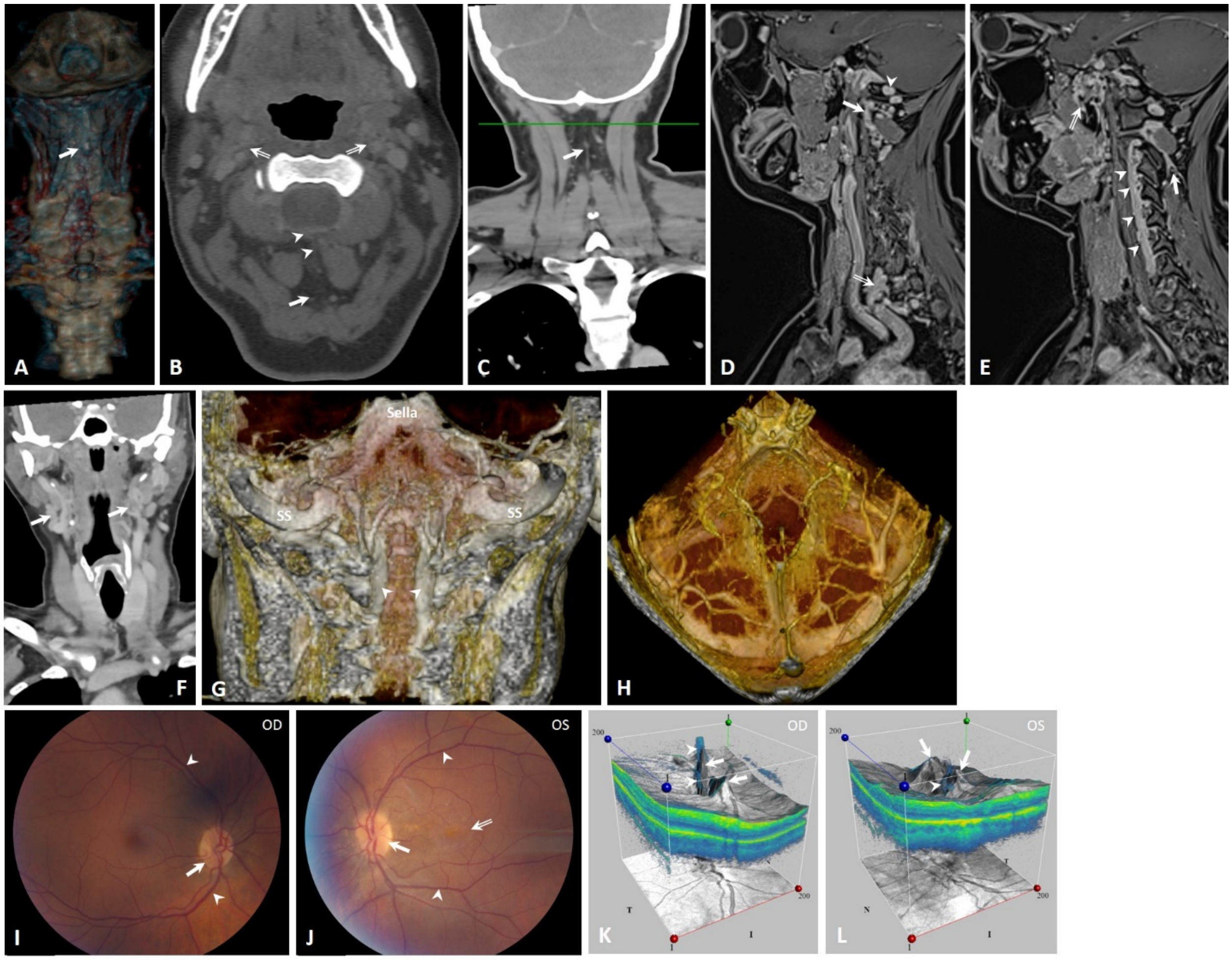

Supplemental Images Patient 7. *Panel A:* 3D volumetric reconstruction of CT of the neck with contrast shows the sub-fascial cavernous malformation (arrow). *Panel B:* Source image axial CT of the neck with contrast shows the malformation (arrow) and drainage to the epidural plexus (arrowheads); rete mirabile is also seen (double-lined arrows). *Panel C:* Source image coronal CT of the neck with contrast shows the malformation (arrow). *Panel D:* Sagittal T1-weighted post-contrast MRI shows the prominent cranio-cervical venous plexus (arrow) draining the posterior condylar emissary vein (arrowhead). Drainage of prominent segmental veins into the plexiform junction of the vertebral vein and subclavian vein is also seen (double-lined arrow). *Panel E:* Sagittal T1-weighted post-contrast MRI shows the prominent vertebral vein (arrowheads) draining the foraminal and segmental veins at each level. Venous drainage of the sub-fascial cavernous malformation to the cranio-cervical plexus is also seen (arrow). A prominent aberrant venous plexus within the pterygopalatine fossa is also seen (double-lined arrow). *Panel F:* Coronal CT of the neck with contrast shows bilateral rete mirabile (arrows). *Panel G:* Volumetric reconstruction of T1-weighted post-contrast MRI of the neck shows cervical venous sinuses (arrowheads). SS, sigmoid sinus. *Panel H:* Volumetric reconstruction of T1-weighted post-contrast MRI of the head shows the venous anatomy of the tentorium. *Panel I:* Fundoscopy of the right eye (OD) with acuity of 20/20 shows optic disc elevation (arrow) without optic cup, abnormal retinal pigment epithelium throughout, and large veins (arrowheads). *Panel J:* Fundoscopy of the left eye (OS) with acuity of 20/40 shows optic disc elevation without optic cup (arrow), abnormality of the macula due to central retinal venous malformation (double-lined arrow), and large veins (arrowheads). There are optic nerve drusen and peripapillary retinal pigment epithelium changes. *Panel K:* 3D visualization of optical coherence tomography of the right eye (OD) shows optic disc elevation (arrows) with fibrovascular membrane originating centrally (arrowheads). *Panel L:* 3D visualization of optical coherence tomography of the left eye (OS) shows optic disc elevation (arrows) with fibrovascular membrane centrally (arrowhead) along the vessels.

eFigure 8.

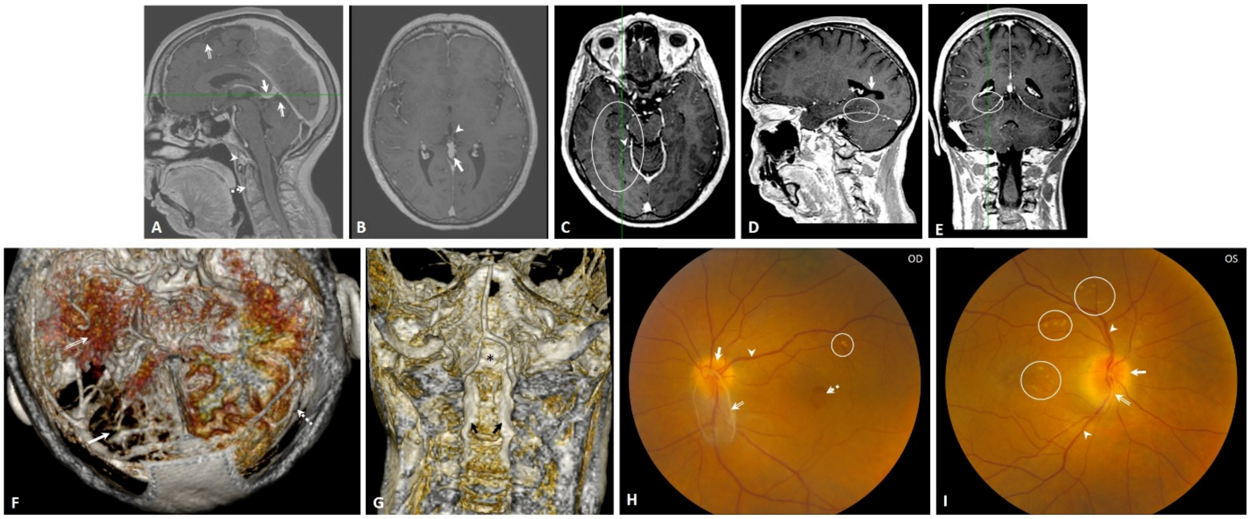

Supplemental Images Patient 8. *Panel A:* Sagittal T1-weighted post-contrast MRI shows a large confluens of the vein of Galen (arrow) and venous phase contrast enhancement within the ligaments of the of the cranio-cervical junction (arrowhead). Contrast enhancement and patent synchondrosis of the dens is also seen (dashed arrow). Prominent subarachnoid spaces (double-lined arrow) and subpial spaces (thick double-lined arrow) are seen. *Panel B:* Axial slice corresponding to the green line in *Panel A* shows the large confluens of the vein of Galen (arrow). Communication between the third ventricle and quadrigeminal cistern is seen (arrowhead). *Panel C:* Axial T1-weighted post-contrast MRI windowed to highlight contrast shows prominent Virchow-Robins spaces (circle) throughout the right temporal lobe with veins arising from a temporal lobe bridging vein to the tentorium (arrowhead). *Panel D:* Sagittal slice corresponding to the green line in *Panel C* shows the tentorially based venous plexus within the temporal lobe (circle). Abnormal morphology of the occipital horn of the lateral ventricle is also seen (arrow). *Panel E:* Coronal T1-weighted post-contrast MRI shows the same prominent veins and Virchow-Robins spaces in the right temporal lobe (circle). *Panel F:* Volumetric reconstruction of T1-weighted post-contrast MRI of the head shows tentorial venous drainage (arrow). Prominent Virchow-Robins or interstitial fluid spaces are shown in red. The right temporal lobe malformation is seen (double-lined arrow). An additional malformation is seen in the right occipital lobe draining into the transverse sinus (dashed arrow). *Panel G:* Volumetric reconstruction of T1-weighted post-contrast MRI of the neck shows dural sinuses (arrows) throughout the upper cervical spine which also drain the mesencephalic plexus (asterisk). *Panel H:* Fundoscopy of the right eye (OD) with acuity of 20/32 shows optic disc elevation without optic cup (arrow) and fibrovascular membrane centrally (double-lined arrow); the veins are large (arrowhead). There is a retinal hemorrhage previously treated with laser coagulation (dashed arrow). There are extra-macular drusen (circle). *Panel I:* Fundoscopy of the left eye (OS) with acuity of 20/100 shows optic disc elevation without optic cup (arrow), fibrovascular membrane (double-lined arrow), and extra-macular drusen (circles); the veins are large (arrowhead).

eFigure 9.

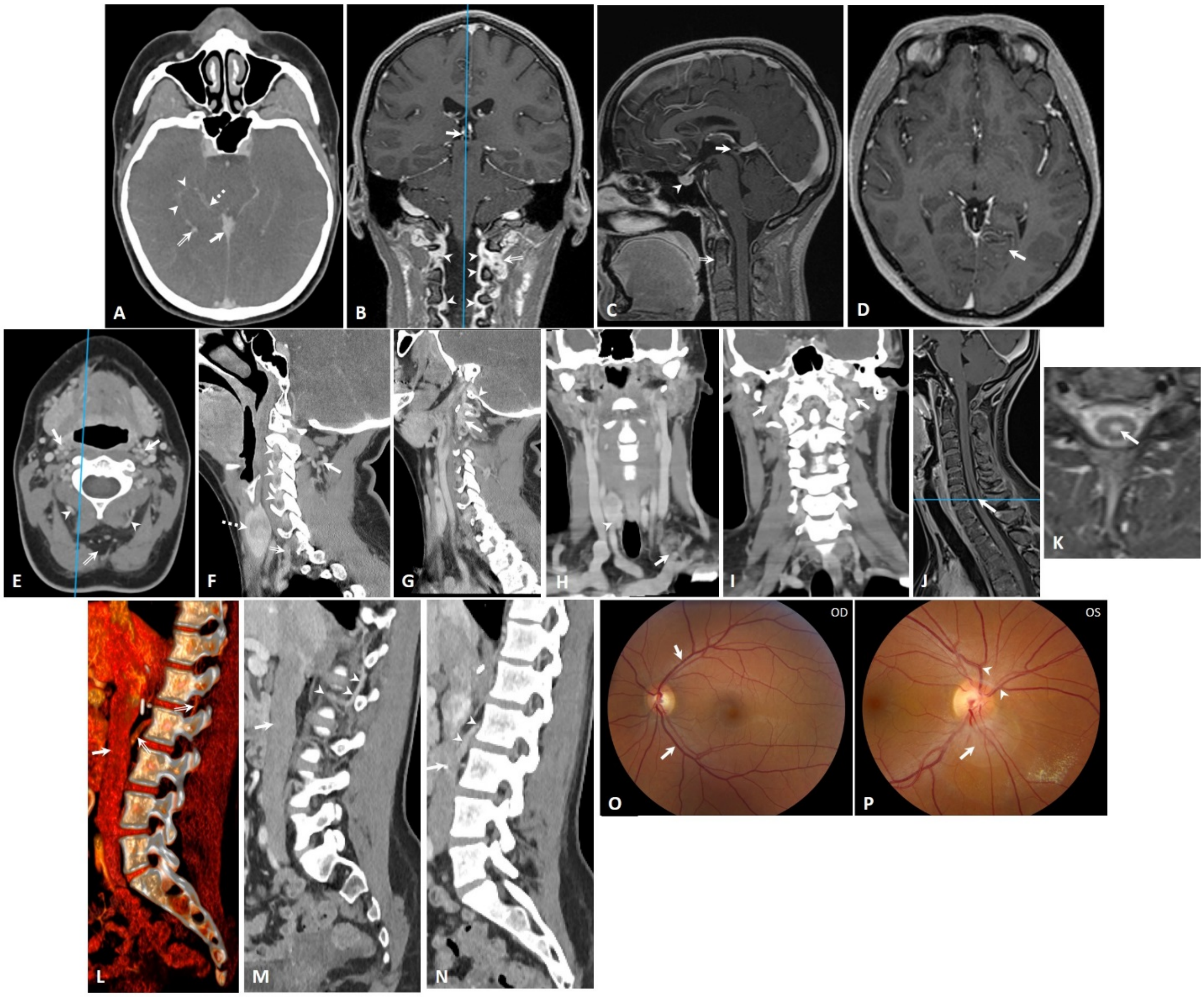

Supplemental Images Patient 9. *Panel A:* Axial delayed phase CTA of the head shows a large vein of Galen (arrow) and a developmental venous anomaly (arrowheads) connecting the right choroid plexus (double-lined arrow) within the lateral ventricle to the basal vein of Rosenthal (dashed arrow); this is present on the left also but not completely visible in this slice. *Panel B:* Coronal T1-weighted post-contrast MRI shows a venous malformation within the quadrigeminal cistern (arrow) and prominent venous cranio-cervical plexus (double-lined arrow) draining through cervical venous sinuses (arrowheads). *Panel C:* Sagittal slice corresponding to the blue line in panel B shows the prominent vein of Galen draining the venous malformation of the quadrigeminal cistern (arrow); the pituitary gland appears mildly hypervascular (arrowhead). Uncalcified synchondroses of the dens are seen (double-lined arrow) with abnormal contrast enhancement. *Panel D:* Axial T1-weighted post-contrast MRI shows a tentorial-based venous malformation of the left occipital lobe (arrow). *Panel E:* Axial delayed phase CTA of the neck shows rete mirabile bilaterally (arrows), prominent veins throughout, and the sub-fascial cavernous malformation (double-lined arrow) draining to segmental veins bilaterally (arrowhead). *Panel F:* Sagittal slice corresponding to the blue line in panel E shows the segmental vein (arrow) draining the cavernous malformation to prominent foraminal veins (arrowheads); these drain to a prominent vertebral vein draining to the subclavian (double-lined arrow). A large hypodensity in the right lobe of the thyroid is seen. *Panel G:* Sagittal slice of the left side of the neck shows a malformation of the cranio-cervical plexus (arrow) arising from the posterior condylar emissary vein (arrowhead). *Panel H:* Coronal slice shows a venous malformation arising from the left subclavian vein (arrow). The hypodensity in the thyroid is again seen (arrowhead). *Panel I:* Coronal slice posterior to panel H shows rete mirabile of the carotid arteries and jugular veins bilaterally (arrows). *Panel J:* Sagittal DIXON post-contrast MRI sequence shows an enlarged central canal (arrow) in

the cervical spine. *Panel K:* Axial STIR MRI sequence shows the enlarged central canal (arrow). *Panel L:* 3D volumetric reconstruction of CT of the abdomen and pelvis with contrast shows anomalous drainage of the epidural venous drainage of the spine (double-lined arrows) to the inferior vena cava (arrow). *Panels M and N:* Source sagittal images for reconstruction. The anomalous drainage of the epidural veins is shown (arrowheads) entering the inferior vena cava (arrow). *Panel O:* Fundoscopy of the right eye (OD) with acuity of 20/20 shows prominent retinal veins (arrows). *Panel P:* Fundoscopy of the left eye (OD) with acuity of 20/20 shows an endophytic juxta-papillary retinal capillary hemangioma within fibrovascular membrane (arrow), and prominent retinal veins (arrowheads).

eFigure 10.

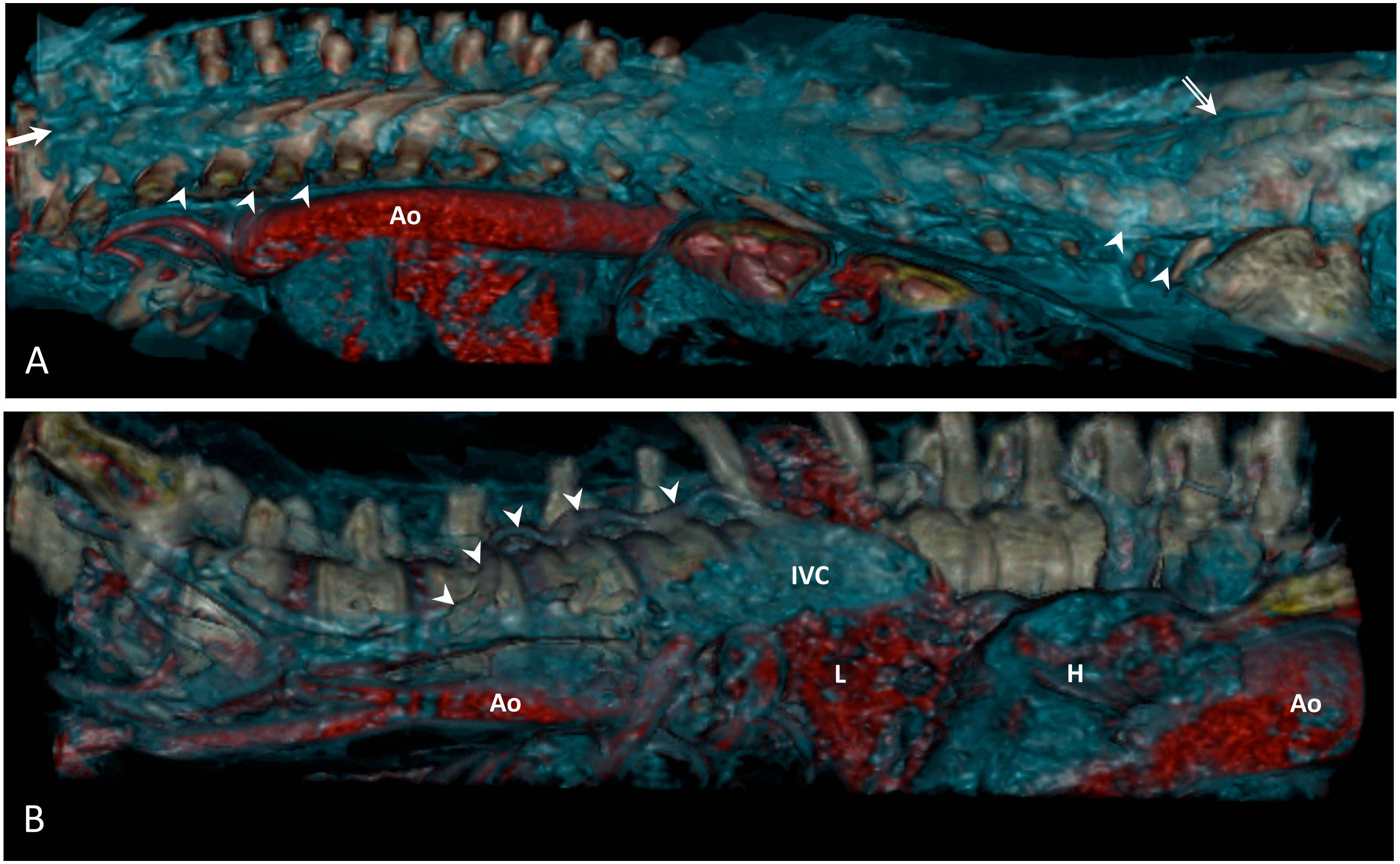

Supplemental Figure 10. Drainage of Systemic Venous Malformations in *EPAS1*-Gain-of-Function Syndrome Patients. *Panel A:* 3D volumetric reconstruction of CT of the chest, abdomen, and pelvis with contrast of patient 1 shows the cervical venous malformation (blue) extending throughout the spine connected by large plexiform intersegmental veins (*e.g.* arrowheads). Veins within the spinal canal (doubled lined arrow) connect to the external spinal venous malformation at the lumbosacral junction through a sacral dysraphism (dashed arrow). Ao, aorta. *Panel B:* The same volumetric reconstruction of CT of the chest, abdomen, and pelvis with contrast shows an aberrant vein (arrowheads) draining the spine venous malformation to the inferior vena cava (IVC). No connection of this draining vessel or the malformations to the aorta (Ao) is seen. The liver (L) and heart (H) are shown for reference.

eFigure 11.

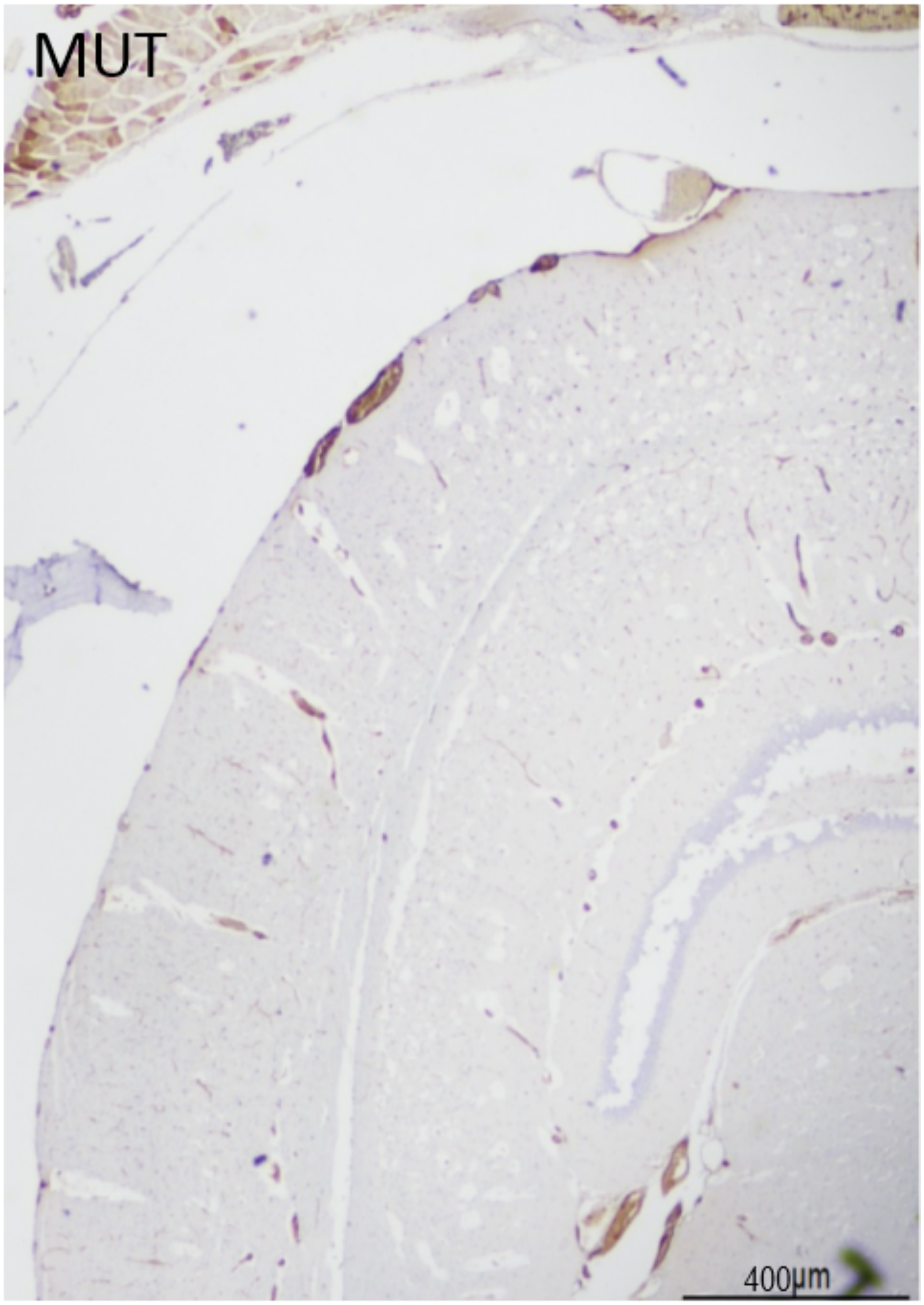

Supplemental Figure 11. Immunohistochemical staining with anti-CD34 antibody in the mutant confirmed the large vessels seen on H&E.

eFigure 12.

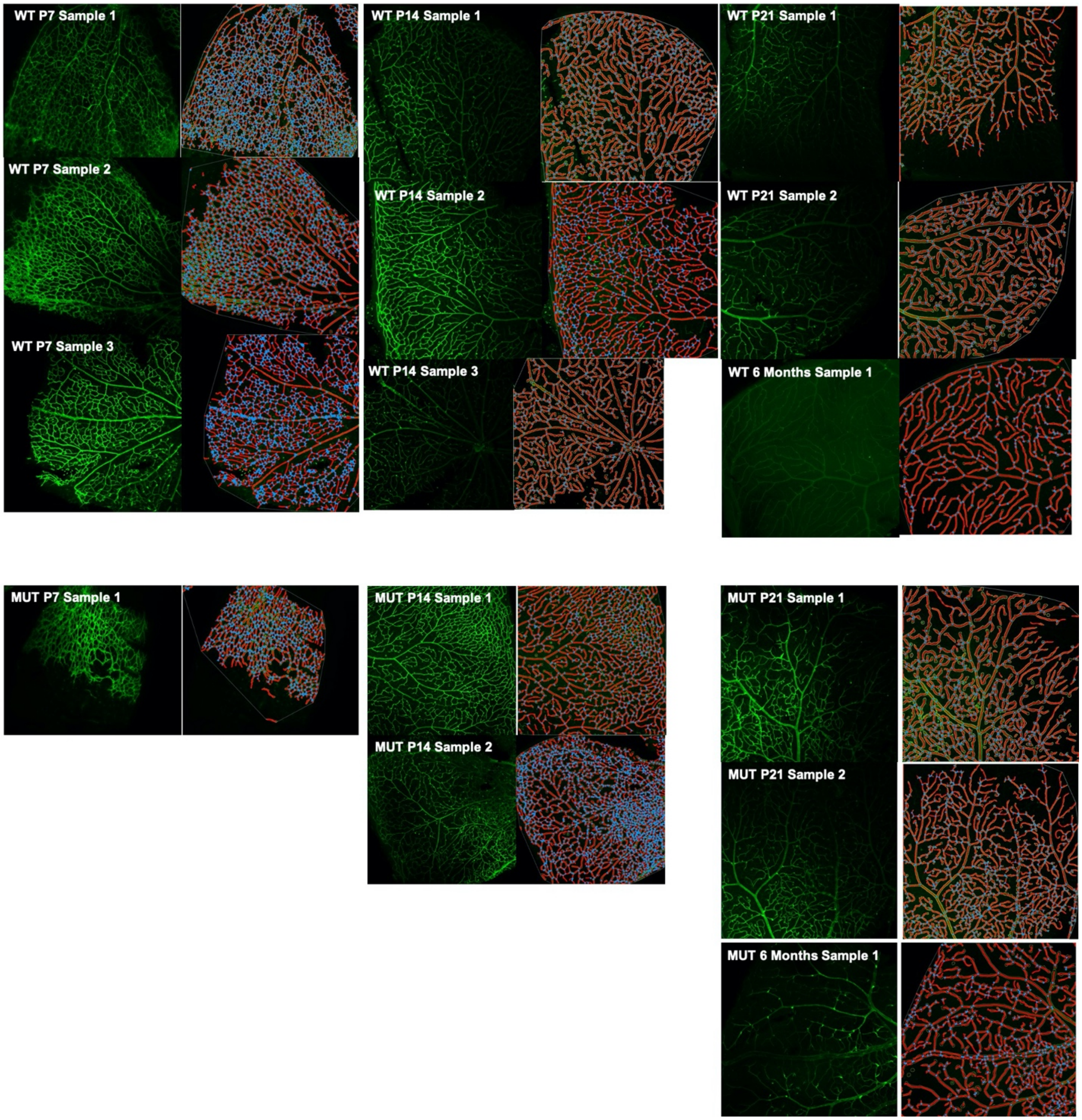

Supplemental Figure 12. Image input (left) and output (right) of each sample from AngioTool (http://angiotool.nci.nih.gov) for quantification of characteristics of the superficial retinal vascular plexus of *EPAS1*-gain-of-function mouse model (MUT) and littermate controls (WT) at post-natal day 7, 14, and 21- and 6-month timepoints.

Supplemental Figure 13.

		Vessels Percentage Area			Junctions density			Mean E Lacunarity		
		Mean	*Double Standard Deviation*	*Standard Error of the Mean*	*Mean*	*Double Standard Deviation*	*Standard Error of the Mean*	*Mean*	*Double Standard Deviation*	*Standard Error of the Mean*
**Timepoint**	**Type**									
**P7 (n=1)**	MUT	31.03	--	--	0.000646	--	--	0.438	--	--
**P7 (n=3)**	WT	34.75	17.69	6.23	0.00181	0.00282	0.000998	0.149	0.0704	0.0249
**P14 (n=2)**	MUT	39.06	8.28	4.14	0.000720	0.000801	0.000400	0.130	0.0210	0.0105
**P14 (n=3)**	WT	29.63	6.30	2.23	0.000751	0.000691	0.000244	0.139	0.0669	0.0237
**P21 (n=2)**	MUT	27.93	6.45	3.23	0.000844	0.000303	0.000151	0.154	0.00337	0.00168
**P21 (n=2)**	WT	24.60	14.50	7.25	0.000590	0.0000462	0.0000231	0.275	0.208	0.104
**P180 (n=1)**	MUT	33.04	--	--	0.000223	--	--	0.184	--	--
**P180 (n=1)**	WT	24.79	--	--	0.000136	--	--	0.248	--	--

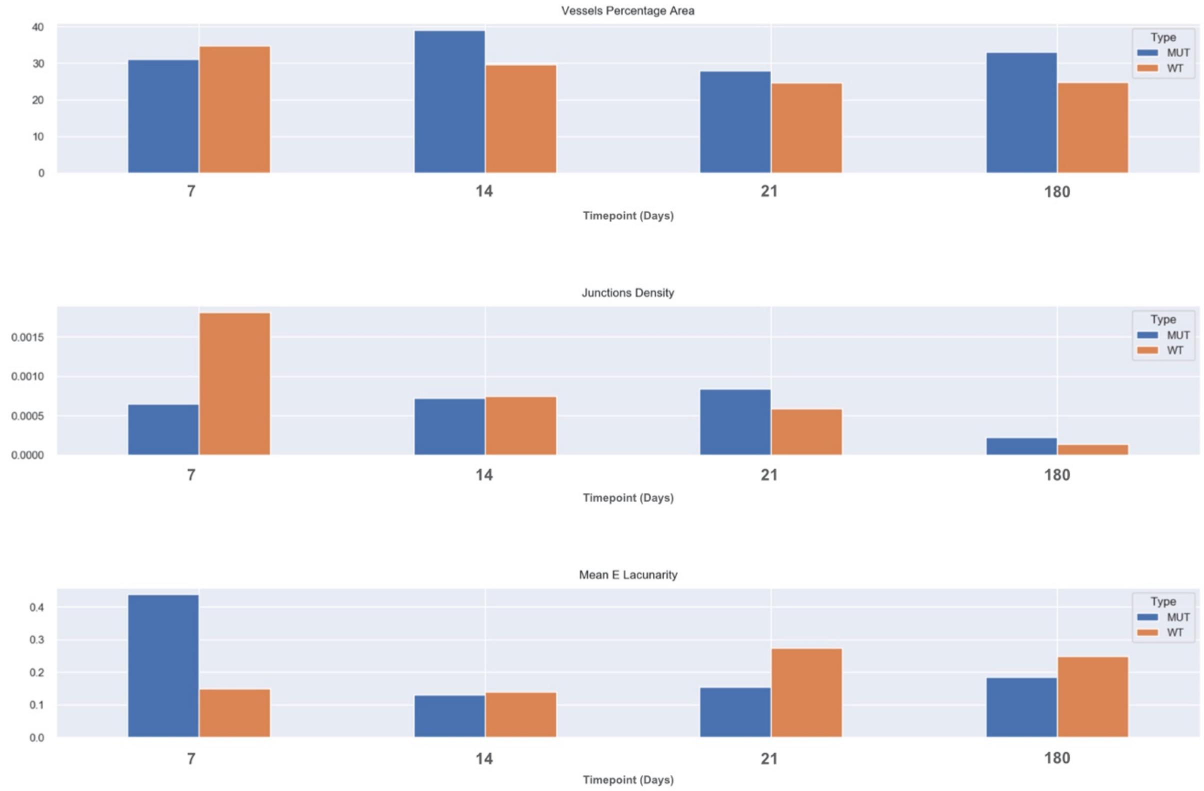

Supplemental Data 1. Data output from quantification of superficial retinal plexus using AngioTool.
